# Emerging new therapeutic antibody derivatives for cancer treatment

**DOI:** 10.1038/s41392-021-00868-x

**Published:** 2022-02-07

**Authors:** Shijie Jin, Yanping Sun, Xiao Liang, Xinyu Gu, Jiangtao Ning, Yingchun Xu, Shuqing Chen, Liqiang Pan

**Affiliations:** 1 Institute of Drug Metabolism and Pharmaceutical Analysis, College of Pharmaceutical Sciences, Zhejiang University, 310058 Hangzhou, China; 2 Department of Precision Medicine on Tumor Therapeutics, ZJU-Hangzhou Global Scientific and Technological Innovation Center, 311200 Hangzhou, China; 3 The First Affiliated Hospital, Zhejiang University School of Medicine, 310003 Hangzhou, China; 4 Key Laboratory of Pancreatic Disease of Zhejiang Province, 310003 Hangzhou, China

**Keywords:** Drug delivery, Drug development

## Abstract

Monoclonal antibodies constitute a promising class of targeted anticancer agents that enhance natural immune system functions to suppress cancer cell activity and eliminate cancer cells. The successful application of IgG monoclonal antibodies has inspired the development of various types of therapeutic antibodies, such as antibody fragments, bispecific antibodies, and antibody derivatives (e.g., antibody–drug conjugates and immunocytokines). The miniaturization and multifunctionalization of antibodies are flexible and viable strategies for diagnosing or treating malignant tumors in a complex tumor environment. In this review, we summarize antibodies of various molecular types, antibody applications in cancer therapy, and details of clinical study advances. We also discuss the rationale and mechanism of action of various antibody formats, including antibody–drug conjugates, antibody–oligonucleotide conjugates, bispecific/multispecific antibodies, immunocytokines, antibody fragments, and scaffold proteins. With advances in modern biotechnology, well-designed novel antibodies are finally paving the way for successful treatments of various cancers, including precise tumor immunotherapy, in the clinic.

## Introduction

Over the past 30 years, therapeutic antibodies have revolutionized the field of targeted cancer therapy. Therapeutic application of monoclonal antibodies (mAbs) emerged after the hybridoma technique to produce mAbs was introduced by Kohler and Milstein in 1975.^1^ The antibody humanization technique pioneered by Greg Winter in 1988 further promoted the development of therapeutic mAbs for treating various cancers.^2^ To date, >100 mAbs have been approved by the US Food and Drug Administration (FDA) for the treatment of different human diseases, including cancer and autoimmune and chronic inflammatory diseases.^3^ mAbs can specifically bind to target antigens and induce cytotoxicity by exerting neutralizing or proapoptotic effects, as well as promote innate immune responses, such as antibody-dependent cellular cytotoxicity (ADCC), complement-dependent cytotoxicity (CDC), and antibody-dependent cellular phagocytosis (ADCP).^4^ Inspired by the successful application of immunoglobulin G (IgG) mAbs, other antibody formats (e.g., antibody fragments, bispecific antibodies (BsAbs), and non-IgG scaffold proteins) and antibody derivatives (e.g., antibody–drug conjugates (ADCs) and immunocytokines) have been successively accepted as alternative therapeutic agents for a broad range of cancers.^5^

The antitumor efficacy of an antibody can be remarkedly improved by linking highly a cytotoxic small molecule to the mAb, generating a novel type of antibody derivative, an ADC.^6^ ADCs can selectively deliver highly cytotoxic small-molecule drugs directly to targeted cancer cells and induce their apoptosis,^7^ which fulfills the requirements of a “magic bullet” as postulated by German physician and scientist Paul Ehrlich more than one century ago.^8^ The FDA has approved 10 ADCs for cancer treatment, and >80 ADCs are under clinical investigation.^9^ In addition to small-molecule drugs, antibodies can be conjugated to other types of molecules, such as oligonucleotides,^10^ radionuclides,^11^ and protein toxins.^12^

Harnessing the power of the human immune system is steadily gaining recognition for its importance in the treatment of cancer.^13^ BsAbs can simultaneously bind to two different antigens.^14^ The most widely used BsAb is a bispecific T cell engager (BiTE), with one arm targeting CD3 on T cells and the other recognizing target proteins on tumor cells, thereby activating the T cells to kill the tumor cells.^15^ One first-in-class BiTE, blinatumomab, which targets both CD19 and CD3, was approved by the FDA for the treatment of patients with relapsed and/or refractory B cell precursor acute lymphoblastic leukemia (R/R B-ALL) in 2014.^16^ In addition to their interaction with T cells, BsAbs have also been designed to engage other effector cells, such as natural killer (NK) cells^17^ and macrophages^18^ for cancer therapy. Antibody–cytokine fusion proteins (also named immunocytokines) represent another novel class of antibody-based immunotherapies.^19^ Cytokines constitute a broad and loosely defined class of relatively small proteins that regulate the immune response.^20^ The systemic administration of proinflammatory cytokines is often associated with severe off-target toxicity, particularly flu-like symptoms, which may limit the dose and prevent the escalation of dosages needed for developing therapeutically effective regimens.^21^ Similar to the ADC strategy, a strategy for using immunocytokines with antibodies or antibody fragments as vehicles has been used for the targeted delivery of immunomodulatory cytokines (such as interleukin (IL)-2, IL-12, and tumor necrosis factor (TNF)) to leverage the local tumor microenvironment (TME) and activate anticancer immune responses.^22^

IgG is the predominant antibody used in current antibody drugs, but in certain cases, the application of full-length antibody is limited in cancer treatment because these large antibodies such as poor penetration into solid tumors and because the Fc can mediate bystander activation of the immune system.^23^ Recent advantages in antibody engineering have facilitated the production of different types of antibody fragments (e.g., Fab, F(ab’)_2_), engineered antibodies (e.g., single-chain variable (scFv) fragments scFabs), and Ig domains (e.g., VHH).^24^ These fragments usually retain the antigen specificity of the full-length antibody and are expected to show better penetration into tumors and fewer Fc-related adverse effects. In addition, non-IgG scaffold proteins, such as affibodies, designed ankyrin repeat proteins (DARPins), and monobodies, represent promising classes of therapeutic and diagnostic molecules.^25^

mAbs usually recognize cell-surface antigens, whereas most cancer-associated proteins reside in intracellular compartments.^26^ T cell receptors (TCRs) can recognize certain small fragments of intracellular proteins by binding with the peptide-major histocompatibility complex (pMHC), which comprises a short peptide derived from intracellular proteins presented in the context of the MHC on the cell surface.^27^ An antibody that mimics the epitope-recognizing segment of a TCR, termed a TCR mimic (TCRm) antibody, and TCRms are being used to target proteins of interest inside tumor cells or other cells.^28^ A TCRm combines the pMHC-targeting ability of a TCR with the robustness of IgG mAbs, which is expected to improve druggability.^29^

In this review, we summarize the advances in the development of new therapeutic antibodies and their applications in cancer treatment.

## Antibody conjugates

### Antibody–drug conjugates

#### Design and structure of ADCs

**Fig. 1:**
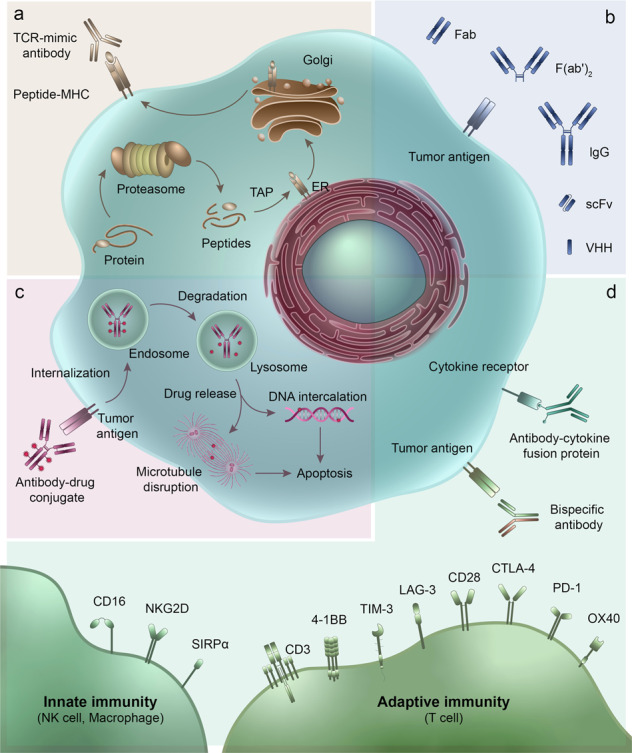
Representative therapeutic antibodies and their derivatives. **a** TCR-mimic antibody; **b** IgG antibody and antibody fragments; **c** antibody-drug conjugate (ADC) and its mechanism of action; **d** multifunctional antibodies, such as bispecific antibodies, immunocytokine (antibody-cytokine fusion protein)

In recent years, the proposed use of ADCs has gradually gained steam, and they are rising stars in the tumor treatment field. An ADC comprises three main components: a mAb, cytotoxic payload, and linker. Upon binding with a target antigen on tumor cells, am ADC can deliver a cytotoxin payload into the targeted cell cytoplasm via receptor-mediated endocytosis, release the cytotoxic drug from the ADC during lysosomal degradation to destroy DNA or otherwise inhibit cell division and eventually kill tumor cells^30^ (Fig. 1c).

Suitable drug targeting, which is highly tumor-specific and readily internalized by cancer cells, is a key factor that determines the druggability of an ADC.^31^ To minimize on-target/off‐target toxicity and open an acceptable therapeutic window for ADC applications, tumor-specific or overexpressed target antigens are preferable for ADC targeting and cytotoxic payload delivery. Ideal ADCs are rapidly and efficiently internalized via the clathrin-mediated pathway and are efficaciously trafficked to lysosomes, where they rapidly accumulate. Currently, more than 50 antigens have been used as targets for the preclinical or clinical development of ADCs; these antigens include human epidermal growth factor receptor 2 (HER2),^32^ trophoblast cell-surface antigen-2 (Trop-2),^33^ and B cell maturation antigen (BCMA).^34^

The cytotoxic payload of an ADC is a highly potent drug capable of efficient cell killing. Compared with the effect of conventional chemotherapy, these payloads showed higher toxic potency (from 100- to 1000-fold). Free ADC payloads cannot be effectively administered as chemotherapy agent candidates due to their extreme potency, but their toxicities can be minimized by directing the potency of the cytotoxic payload by conjugating it to a tumor-specific antibody. ADC payloads can be classified into two major types: (i) tubulin inhibitors inhibit tubulin polymerization and trigger cell cycle arrest in the G2/M phase and subsequent cell apoptosis; these inhibitors include monomethyl auristatin E (MMAE),^35^ monomethyl auristatin F (MMAF),^36^ and a derivative of maytansine 1 (DM1).^32^ (ii) DNA-damaging agents bind the minor groove in DNA, leading to cell death via DNA cleavage, DNA alkylation or interrupted DNA replication (these agents include calicheamicin,^37^ SN-38,^33^ DXd,^38^ and PBD^39^). Other small-molecule payloads, such as α‐amanitin (a selective RNA polymerase II inhibitor), are also under investigation.^40^

Linkers, which covalently conjugate the cytotoxic payloads to the antibody, are also essential components of ADCs. Ideal linkers are stable before they reach the targeted tumor site and are rationally designed for rapid liberation of payloads from an ADC upon entry into lysosomes. Based on the mechanism of payload release, linkers can be categorized into cleavable or noncleavable linkers. Cleavable linkers are designed to conditionally respond to the TME or intracellular environment, such as low pH (e.g., the acid-labile hydrazone-based linker in gemtuzumab ozogamicin (GO)^41^), proteolysis (e.g., the valine-citrulline linker in brentuximab vedotin (BV)^42^), or high-glutathione concentrations (e.g., the disulfide linker in the maytansinoid-based ADC mirvetuximab soravtansine^43^). On the other hand, noncleavable linkers (e.g., the thioether linker in ado-trastuzumab emtansine) rely on complete lysosomal degradation of the antibody for payload release.^44^ The chemical conjugation strategies of ADCs play a significant role in the therapeutic potential of ADCs. Usually, an ADC payload is conjugated to a surface lysine or cysteine residue of an antibody, resulting in the patterned distribution of ADCs with different drug-to-antibody ratios (DARs) during chromatographical separation. Different DARs, which may vary from zero to eight, indicate different ADC pharmacokinetics, efficacy, and safety profiles.^7^ Hence, site-specific conjugation approaches are being explored to generate homogeneous ADCs.^45^

#### Clinical results obtained with approved ADCs

**Fig. 2:**
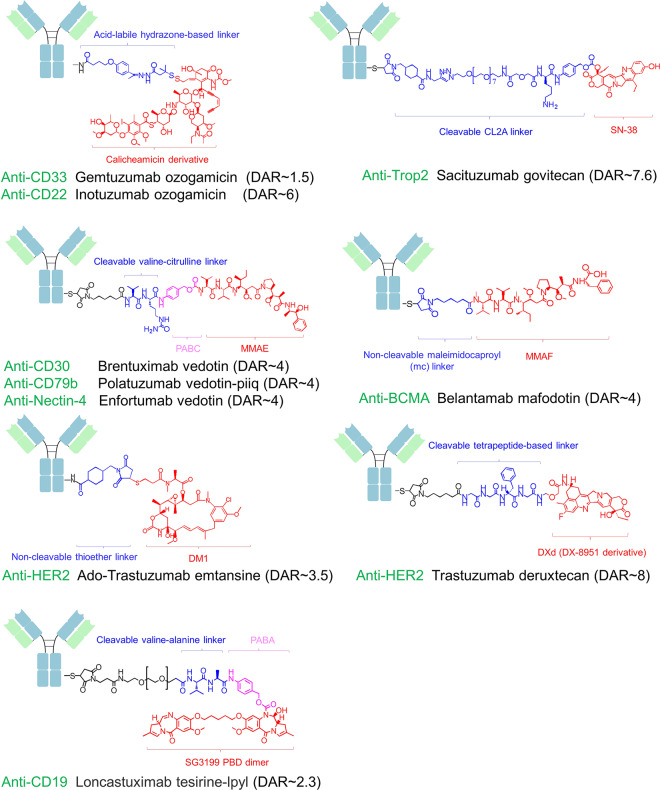
Schematic representation of the FDA-approved antibody-drug conjugate (ADC)

To date, ten ADCs have been approved by the FDA for cancer treatment. An overview of these ADCs, including ADC design and indications, is presented in Table 1 and Fig. 2. A detailed discussion of these FDA-approved ADCs is presented below. ADCs at phase III clinical trial are summarized in Table 2.

**Table 1:** The FDA-approved antibody–drug conjugates

Name	Target	Linker	Payload	Indication(s)	Year of FDA approval
Gemtuzumab ozogamicin (Mylotarg)	CD33	Acid-labile hydrazone-based linker	Calicheamicin derivative	AML	2000; withdrawn in 2010; reapproved in 2017
Brentuximab vedotin (Adcetris)	CD30	Cleavable valine-citrulline linker	MMAE	HL, sALCL	2011
Ado-Trastuzumab emtansine (Kadcyla)	HER2	Non-cleavable thioether linker	DM1	HER2-positive breast cancer	2013
Inotuzumab ozogamicin (Besponsa)	CD22	Acid-labile hydrazone-based linker	Calicheamicin derivative	R/R B-ALL	2017
Polatuzumab vedotin-piiq (Polivy)	CD79b	Cleavable valine- citrulline linker	MMAE	R/R DLBCL	2019
Enfortumab vedotin (Padcev)	Nectin-4	Cleavable valine-citrulline linker	MMAE	Advanced urothelial cancer	2019
Trastuzumab deruxtecan (Enhertu)	HER2	cleavable tetrapeptide-based linker	DXd (DX-8951 derivative)	HER2-positive breast cancer	2019
Sacituzumab govitecan (Trodelvy)	Trop-2	Cleavable CL2A linker	SN-38	TNBC	2020
Belantamab mafodotin (Blenrep)	BCMA	Non-cleavable maleimidocaproyl (mc) linker	MMAF	R/R multiple myeloma	2020
loncastuximab tesirine-lpyl (Zynlonta)	CD19	Cleavable valine-alanine linker	SG3199 (PBD dimer)	R/R DLBCL	2021

**Table 2:** Antibody-drug conjugates at phase III clinical trial

Name	Target	Payload	Indication(s)	ClinicalTrials.gov identifier
Mirvetuximab soravtansine (IMGN853)	Folate receptor α	DM4	Epithelial ovarian cancer, peritoneal cancer, fallopian tube cancer, ovarian cancer	NCT04296890, NCT04209855, NCT02631876
Transtuzumab duocarmazine (SYD985)	HER2	seco-DUBA	Metastatic breast cancer	NCT03262935
Depatuxizumab mafodotin (ABT-414)	EGFR	MMAF	Glioblastoma, gliosarcoma	NCT02573324, NCT03419403
Disitimab vedotin (RC48-ADC)	HER2	MMAE	Locally advanced or metastatic breast cancer with low expression of HER2	NCT04400695

#### Gemtuzumab ozogamicin

The FDA approved the first ADC drug—GO (Mylotarg^®^, Pfizer/Wyeth)—in 2000 for the treatment of patients aged >60 years with relapsed CD33-positive acute myeloid leukemia (AML).^46^ GO is a CD33-directed ADC consisting of recombinant humanized IgG4 antibody specific to CD33, a calicheamicin derivative, and an acid-labile hydrazone-based linker that covalently attaches the toxin to the antibody.^37^ However, in a postmarket clinical trial, GO failed to show improvement in complete response (CR), overall survival (OS), or disease-free survival; in contrast, an increase in treatment-related mortality was shown.^47^ Therefore, Pfizer withdrew the anti-CD33 ADC Mylotarg from the market in 2010.

In September 2017, on the basis of results from 3 clinical trials (ALFA-0701,^48^ AML-19,^49^ and MyloFrance-1^50^), the FDA reapproved GO for patients newly diagnosed with CD33-positive ALL and for patients 2 years and older with relapsed or refractory CD33-positive AML. A phase III ALFA-0701 study compared standard front-line chemotherapy (daunorubicin and cytarabine) with and without a low fractionated dose of GO for patients with untreated de novo AML. The results showed that event-free survival (primary endpoint) was longer for patients in the GO group than for those in the chemotherapy-alone group (median 15.6 vs. 9.7 months; hazard ratio (HR), 0.58; 95% confidence interval (CI), 0.43–0.78; *p* = 0.0003). increased OS (median 34.0 vs. 19.2 months; HR 0.69; 95% CI, 0.49–0.98; *p* = 0.0368) and relapse-free survival (median 28.1 vs. 11.4 months; HR, 0.52; 95% CI, 0.36–0.75; *p* = 0.0003) were also observed. In the GO group, there was no increase in the risk of death from toxicity, although severe adverse events were more frequent. The randomized phase III AML-19 trial was performed to evaluate GO monotherapy and compare the results with that of the best supportive care (BSC) used for treating elderly patients with previously untreated AML. Patients assigned to the GO group exhibited a significantly longer OS than patients in the BSC group (median 4.9 months vs. 3.6 months; HR, 0.69; 95% CI, 0.53–0.90; *p* = 0.005). In a third trial, MyloFrance-1, a phase 2, single-arm, open-label study, included 57 patients with CD33-positive AML in their first relapse. Patients received GO at a dose of 3 mg/m^2^ on Days 1, 4, and 7. Fifteen (26%; 95% CI, 16–40%) patients achieved CR with a median relapse-free survival of 11.6 months. Both AML-19 and MyloFrance-1 showed the effectiveness and safety of GO administered as a single drug.

Ongoing studies are being performed to investigate the treatment of AML on the basis of various GO dosing schedules and in combination with chemotherapy.

#### Brentuximab vedotin

BV (Adcetris^®^, Seattle Genetics, Inc.) is an ADC composed of a chimeric anti-CD30 IgG1 antibody covalently linked to the microtubule-disrupting small-molecule MMAE via a cleavable valine-citrulline linker.^51^ In 2011, BV was approved by the FDA to treat patients with systemic anaplastic large-cell lymphoma (ALCL) and relapsed or refractory CD30-positive Hodgkin lymphoma (HL). Approval was based on two single-arm phase II trials.

ALCL is an aggressive subtype of T cell lymphoma characterized by uniform expression of CD30. In a single-arm phase II multicenter trial, patients with relapsed or refractory systemic ALCL after at least one prior therapy were treated with BV 1.8 mg/kg intravenously every 3 weeks. The trial showed an OR of 86% and a CR of 57%.^52^ For patients with relapsed or refractory HL (R/R HL), a phase II study showed that BV was associated with manageable toxicity and high efficacy. Of the 102 patients treated in the study, 76 patients (75%) achieved an objective response, 35 patients (34%) achieved complete remission, and 41 patients (40%) achieved partial remission. Grade 1 or 2 adverse effects were the most common; they included peripheral sensory neuropathy, nausea, fatigue, neutropenia, and diarrhea.^53^

Cutaneous T cell lymphomas (CTCLs) are rare non-HL subtypes that involve the skin and blood, lymph nodes, and other internal organs. In 2017, the FDA approved BV to treat adult patients with CD30^+^ CTCL, including patients with primary cutaneous ALCL (pcALCL) and mycosis fungoides who had been previously treated. Approval for the use of BV was based on the phase III ALCANZA trial, which showed a significant increase in an objective response lasting at least 4 months after BV treatment compared to the objective response to a physician’s choice of either methotrexate or bexarotene (56.3 vs. 12.5%, respectively; 95% CI, 29.1–58.4; *p* < 0.0001).^54^

BV is also effective when used in combination with chemotherapy. Based on results from the ECHELON-1 and ECHELON-2 trials, BV showed potent single-agent activity, and BV in combination with chemotherapy agents was shown to be more effective than chemotherapy alone.^55,56^ Hence, the FDA expanded approval of BV in combination with chemotherapy for the first-line treatment of stage III or IV classical HL or for previously untreated systemic ALCL or other CD30-expressing peripheral T cell lymphomas.

Currently, 78 active (phases I–III) clinical trials registered at ClinicalTrials.gov are being conducted to evaluate BV as a treatment for patients with hematological malignancies.

#### Ado-trastuzumab emtansine

HER2 is overexpressed in approximately 20% of breast cancer patients.^57^ In 2013, Ado-trastuzumab emtansine (also known as T-DM1 or Kadcyla^®^, Genentech, Inc.), a HER2-targeting ADC incorporating the anti-HER2 trastuzumab with the microtubule inhibitor DM1 (a maytansine derivative) via a stable thioether linker, was approved for the treatment of patients with HER2-positive metastatic breast cancer.^58^ The approval was based on a phase III trial (EMILIA).^59^ In the EMILIA trial, patients were randomly administered either T-DM1 (*n* = 495) or lapatinib plus capecitabine (*n* = 496). Median progression-free survival (PFS) (30.9 vs. 25.1 months; HR, 0.68; 95% CI, 0.55–0.85; *p* < 0.001) and median OS (9.6 vs. 6.4 months; HR, 0.65; 95% CI, 0.55–0.77; *p* < 0.001) were significantly better in the patients who received T-DM1 than in the patients who received lapatinib plus capecitabine.

In addition to breast cancer, T-DM1 has been studied in patients with other solid tumors, including lung, bladder, brain, and colorectal cancer.

#### Inotuzumab ozogamicin

The FDA licensed inotuzumab ozogamicin (BESPONSA^®^, Wyeth/Pfizer) for the treatment of patients with R/R B-ALL in 2017. Inotuzumab ozogamicin is an ADC composed of a recombinant humanized IgG4 mAb against CD22 covalently linked to the DNA-damaging agent calicheamicin via an acid-labile hydrazone-based linker.^60^ In a phase III trial (INO-VATE), the effects of inotuzumab ozogamicin treatment were compared with standard intensive chemotherapy in ALL patients, and the results showed that the CR was 81% for the inotuzumab ozogamicin group and 29% for the standard therapy group.^61^ Improvements in PFS (median, 5.0 months vs. 1.8 months; HR, 0.45; 95% CI, 0.34–0.61; *p* < 0.001) and OS (7.7 months vs. 6.7 months; HR, 0.77; 95% CI, 0.58–1.03; *p* = 0.04) were also observed.

Currently, 24 ongoing clinical trials (phases I-III registered at ClinicalTrials.gov) are being performed to test the effect of inotuzumab ozogamicin for treating ALL patients.

#### Polatuzumab vedotin-piiq

Polatuzumab vedotin-piiq (POLIVY^®^, Genentech, Inc.) is a CD79b-specific ADC that consists of a humanized anti-CD79b IgG1 antibody, MMAE, and a cleavable valine-citrulline linker that covalently conjugates MMAE to the polatuzumab antibody.^62^ In June 2019, the FDA granted accelerated approval to polatuzumab vedotin-piiq for adult patients with relapsed or refractory diffuse large B cell lymphoma (DLBCL), which is the most common type of non-Hodgkin lymphoma (NHL).^63^ Approval was based on a multicenter, open-label study (NCT02257567), and on the basis of the study design, polatuzumab vedotin-piiq was administered in combination with bendamustine (B) and rituximab (R).^64^ In this study, polatuzumab vedotin administered in combination with BR resulted in a significantly increased CR (40% vs. 17.5%; *p* = 0.026), median PFS (9.5 vs. 3.7 months; HR, 0.36; 95% CI, 0.21–0.63; *p* < 0.001) and median OS (12.4 months vs. 4.7 months; HR, 0.42;, 95% CI, 0.24–0.75; *p* = 0.002) compared with BR combination therapy without polatuzumab vedotin.

Twenty clinical trials have been recently established for studying polatuzumab vedotin-piiq for the treatment of patients with NHL or DLBCL.

#### Enfortumab vedotin

Enfortumab Vedotin (Padcev^®^, Astellas Pharma US, Inc.), also known as ASG-22ME, is a first-in-class ADC directed against Nectin-4 (Poliovirus receptor-related 4; PVRL4), which is highly expressed in urothelial carcinoma as well as breast, gastric, and lung cancers.^65^ Urothelial cancer, which typically occurs in the urinary system, is the most common type of bladder cancer.^66^ In December 2019, enfortumab vedotin (PADCEV, Astellas Pharma US, Inc.) was granted accelerated approval from the FDA for treating patients with locally advanced or metastatic urothelial cancer and who had been previously treated with anti-PD-1 (programmed cell death-1)//PD-L1 (programmed cell death-1 ligand 1) therapy or platinum-containing chemotherapy. The clinical basis for the FDA approval was the phase II single-arm trial EV-201.^67^ For patients with metastatic urothelial cancer, 55 (44%) of 125 patients showed an objective response, the median duration of the response was 7.6 months, and 15 (12%) patients showed a CR.

Enfortumab vedotin is currently being evaluated for its effect on urothelial cancers of various stages in eight ongoing clinical trials and for its effect on breast, lung, and other solid tumors in three other ongoing clinical studies, according to the registration information at ClinicalTrials.gov.

#### Trastuzumab deruxtecan

Trastuzumab deruxtecan (also known as DS-8201, ENHERTU®, AstraZeneca and Daiichi Sankyo) is an ADC composed of anti-HER2 trastuzumab, a cleavable tetrapeptide-based linker (GGFG), and a potent topoisomerase I inhibitor (an exatecan derivative, DXd) as the payload.^68^ On the basis of two key clinical trials, trastuzumab deruxtecan was granted accelerated approval by the FDA in 2019 for the treatment of patients with unresectable or metastatic breast cancer and who had been treated with at least two prior anti-HER2 regimens in the metastatic context. The phase I trial (NCT02564900) evaluated the safety and tolerability of trastuzumab deruxtecan in patients with HER2-positive advanced-stage breast cancer and with previously administered T-DM1 treatment. Trastuzumab deruxtecan showed a manageable safety profile and potent preliminary activity.^69^ In the second clinical trial (NCT03248492), the ORR was 60.9% (95% CI, from 53.4 to 68.0), and the median response duration was 14.8 months (95% CI, from 13.8 to 16.9). The median PFS was 16.4 months (95% CI, from 12.7 with an unreached maximum).^70^

Encouraged by the results of these clinical trials, approximately 29 clinical trials are currently aimed at evaluating trastuzumab deruxtecan as a treatment for patients with HER2-positive solid tumors, as reported at ClinicalTrials.gov.

#### Sacituzumab govitecan

Triple-negative breast cancer (TNBC) cells lack estrogen receptor, progesterone receptor or HER2 expression, limiting their response to hormonal therapy or HER2-targeted therapies.^71^ Patients with TNBC have limited treatment options. Sacituzumab govitecan (TRODELVY®, Immunomedics, Inc.) is an FDA-approved ADC that incorporates a humanized anti-Trop-2 mAb (hRS7), a cleavable linker, and the topoisomerase 1 inhibitor SN-38 as the payload.^72^ Compared to the payloads of other FDA-approved ADCs (pM), the potency of the SN-38 payload is moderate (at the single-digit nM level).^73^ Owing to the novel linker technology (a polar PEG-based linker), sacituzumab govitecan achieved a relatively high DAR (~7.6) in comparison with currently administered ADCs (DAR = ~4). Sacituzumab govitecan was approved by the FDA on an accelerated basis for refractory metastatic TNBC in April 2020 based on positive results from one clinical trial of 108 patients with TNBC (NCT01631552);^74^ in this clinical trial, the response rate was 33.3%, and the median duration of the response was 7.7 months. Sacituzumab govitecan is an important advancement for the treatment of patients, as it is the first approved ADC specifically targeted to metastatic TNBC.

Phase III trials of sacituzumab govitecan for treating patients with TNBC (NCT04595565), metastatic breast cancer (NCT04639986, NCT03901339) and other solid tumors (NCT04319198, NCT04527991) are ongoing.

#### Belantamab mafodotin

BCMA plays a central role in multiple myeloma (MM) pathogenesis in vivo and is overexpressed in MM cells. Therefore, BCMA has been shown to be a promising cell surface antigen for targeted therapies, such as CAR-T therapy, BsAbs, and ADCs, improving the landscape for patients with relapsed or refractory multiple myeloma (RRMM).^75^ Belantamab mafodotin (BLENREP®, GlaxoSmithKline) is a first-in-class anti-BCMA ADC that was granted accelerated approval by the FDA in August 2020 as a monotherapy treatment for adult patients with RRMM who had received at least four prior therapies; belantamab mafodotin includes an anti-CD38 mAb, the proteasome inhibitor MMAF and a non-cleavable maleimidocaproyl (mc) linker.^76^ The approval was based on results from a two-arm randomized open-label phase II study (DREAMM-2).^77^ In this trial, 97 patients were intravenously administered the recommended dose of 2.5 mg/kg belantamab mafodotin once every 3 weeks. The ORR was 31% (97.5% CI, 21%-43%), and 73% of the responders showed improvement that persisted for 6 months or longer. However, belantamab mafodotin can cause serious eye problems, including corneal changes, decreased vision, and/or blurred vision.

Clinical trials of belantamab mafodotin administered as a monotherapy and in combination with chemotherapy are ongoing for treating patients with MM.

#### Loncastuximab tesirine-lpyl

CD19 is widely expressed during various stages of B cell development and differentiation from pre-B cells to plasma cells, and it is an attractive target for treating B cell malignancies.^78^ Loncastuximab tesirine-lpyl (ZYNLONTA®, ADC Therapeutics SA) is a novel anti-CD19 ADC composed of a humanized anti-CD19 antibody, the alkylating agent pyrrolobenzodiazepine (PBD) dimer SG3199, and a cathepsin-cleavable valine-alanine linker.^79^ On April 23, 2021, loncastuximab tesirine-lpyl was granted accelerated approval by the FDA as a monotherapy treatment for adult patients with relapsed or refractory DLBCL who had received at least two prior systemic therapies.^80^ The approval was based on phase II multicenter open-label and single-arm LOTIS-2 trial (NCT03589469),^81^ in which a total of 145 patients were enrolled. Loncastuximab tesirine-lpyl showed an ORR of 48.3% (95% CI, 39.3–56.7), and the median duration of the response was 10.3 months (95% CI, from 6.9 to an inestimable value). PBD is approximately 50–100-fold more toxic than other cytotoxic ADC payloads,^39^ but loncastuximab tesirine-lpyl with PBD as the payload showed an acceptable safety profile. In the LOTIS-2 trial, the most common (≥10%) grade ≥3 adverse effects including laboratory-measured abnormalities were neutropenia (26.2%), thrombocytopenia (17.9%), an increased gamma-glutamyltransferase (17.2%) level, and anemia (10.3%).

Currently, loncastuximab tesirine-lpyl is under investigation for the treatment of various lymphomas as a single agent or in combination therapy in four active phase I–III clinical trials (ClinicalTrials.gov).

#### Antibody–small interfering RNA (siRNA) conjugates (ARCs)

**Fig. 3:**
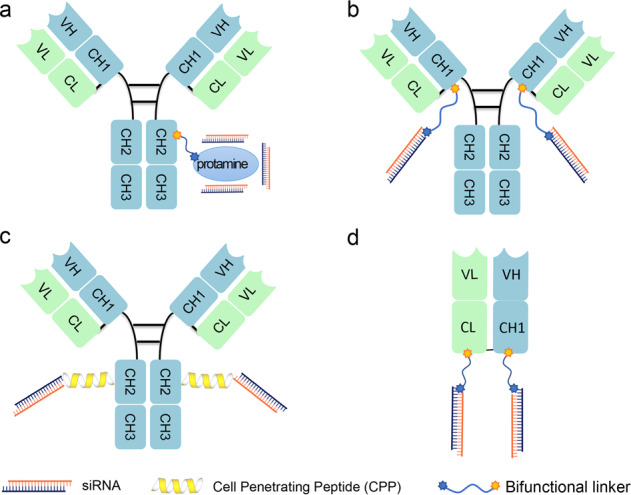
Schematic representation of antibody-siRNA conjugates (ARCs). **a** Antibody siRNA complex is constructed using electrostatic non-covalent interactions. **b** The THIOMAB-based ARC. The siRNA is chemically conjugated with the introduced cysteine of IgG antibody (THIOMAB, Genentech Inc) via thiol-maleimide reaction to achieve site-specific conjugation. **c** IgG-based ARC that incorporates cell-penetrating peptide in the linker to facilitate endosomal escape of siRNA. **d** Fab-based ARC. The siRNA or ASO is chemically conjugated to the C-terminus of Fab

As of January 2021, four siRNA-based drugs with lipid nanoparticle (LNP) or *N*-acetylgalactosamine (GalNAc) delivery systems had received regulatory approval from the FDA or European Medicines Agency (EMA).^82^ Encapsulated in LNPs or covalently conjugated to GalNAc ligands, siRNAs can be successfully delivered to the liver. Although RNA interference-based therapies are enabling progress in RNA delivery to the liver, low delivery efficiency and limited target organs (namely, the liver and eye) appear to be major obstacles for targeted siRNA therapeutics. Since antibodies show high specificity and affinity toward overexpressed antigens in certain cell types or tissues, they are drawing increasing attention as suitable vehicles for siRNA delivery. It has been sixteen years since antibodies were first used to mediate the in vivo delivery of siRNAs via cell-surface receptors.^83^ Through noncovalent interactions with antibody–fusion molecules or covalent linkages with lysine or cysteine residues, siRNAs can be coupled with antibodies for treating cancer (breast cancer,^84^ prostate cancer,^85^ colon cancer,^86^ and MM^87^) or other diseases (HIV^83^ and leukemia^88^). A schematic diagram of ARCs is shown in Fig. 3.

In late 2015, using its THIOMAB platform, Genentech achieved site-specific, large-scale siRNA conjugation to antibodies.^89^ However, the results indicated that the entrapment of ARC in endosomes was a major limiting factor for its further application. Recently, Alnylam Pharmaceuticals, which had previously developed three siRNA drugs (i.e., patisiran, givosiran, and lumasiran) that were approved by the FDA, reported the simple generation of a structurally defined ARC (DVD-ARC) without introducing mutations or using enzymes.^86^ Moreover, the reported ARCs significantly downregulated the target mRNA and protein expression levels in tumor cells, further demonstrating the rationale of using antibodies as vehicles to specifically deliver siRNAs to nonliver tissues. In the late 2010s, Avidity Biosciences described an IgG-based antibody-siRNA conjugate strategy that achieved significant mRNA silencing in muscles and tumors, according to its published patent.^90^ For the development of antibody–oligonucleotide conjugates (AOCs), the Avidity AOC platform enables coupling of various types of oligonucleotides, including siRNAs and ASOs, to IgG antibodies. AOC 1001, a leading AOC drug candidate for treating adult patients with DM1, will enter a phase I/II clinical trial in 2021. This ARC comprises a mAb targeting transferrin receptor 1 (TfR1), a bridging motif (a bifunctional linker with or without a cell-penetrating peptide), and a rationally designed siRNAs to specifically target certain mRNAs. In addition to muscle disease, ARC applications have been expanded to other cell types, such as immune cells and heart cells, according to one manufacturer’s website (Avidity Bioscience). In addition to an IgG-based ARC strategy, using antigen-binding fragments (Fabs) as delivery vehicles has also been extensively explored.^91^ Due to the lack of a Fc domain, Fabs offer multiple powerful advantages over mAbs, including increased tissue penetration and tolerance, enhanced tissue penetration, and reduced risk of immune system activation.^92^ Dyne Therapeutics is pioneering the development of Fab-siRNA/ASO conjugates through its FORCE platform to treat rare muscle diseases, such as DM1, Duchene muscular dystrophy, and facioscapulohumeral muscular dystrophy. The FORCE platform targets TfR1, which is highly expressed on the surface of muscle cells,^93^ enabling efficient and targeted delivery of siRNAs or ASOs directly to skeletal, cardiac, and smooth muscle tissue.

However, the applications of ARCs in the cancer treatment field are still at an early stage, awaiting further exploration. There are several issues in using ARCs that remain unsolved. First, ARCs do not readily enter cells because the negative charge of the appended siRNA makes it difficult to overcome the thermodynamic barriers presented by the cell membrane.^94^ Second, similar to other delivery systems, endosomal escape is a major obstacle for intracellular delivery of siRNAs, leading to inefficient localization of siRNAs into the cytoplasmic RISC.^95^ Third, the inherent endocytic property of the targeted antigen determines the efficiency of siRNA delivery.^90^ For example, Fab-type ARCs have only been shown effective against the target antigen TfR1,^91^ a receptor prevalent in the endocytic pathway. Fourth, the lack of quantitative approaches for investigating the endosomal escape of siRNAs (released from ARCs) and the interaction of siRNAs with the RISC machinery further limits the rational design and optimization of ARCs.^87^ Finally, steric hindrance created during the conjugation of antibodies with a siRNA lowers conjugation efficiency. With the current technology of the ARC field, the ADC linker cannot simply be simply grafted into the conjugate, suggesting that extensive linker optimization is needed to generate certain ARCs. With the boom in ADC production, the strategic use of ARCs, which is similar to the rationale of ADC use, is expected to create new opportunities for the targeted interference of gene expression in multiple organs in vivo in the near future.

## Multispecific antibodies

In the past three decades, multispecific antibodies have rapidly received tremendous attention as therapeutic agents to address unmet clinical needs.^14,96^ Multiple mediators contribute to the activation of cancer-related signaling pathways in the complex pathogenesis of cancer, which limits the efficacy of monospecific-based cancer treatment.^97,98^ A decrease in activated lymphocytes in the TME has been demonstrated to result in an unfavorable immune response.^99,100^ A broad variety of multispecific antibody formats has been developed to function through different mechanisms in cancer immunotherapy, including but not limited to (1) engaging T cells or other immune cells (e.g., NK cells) to specifically eliminate tumor cells, (2) bridging receptors to block or activate synergistic signaling pathways, and (3) targeting multiple tumor antigens or different antigen epitopes on tumor cells to increase tumor selectivity (Fig. 1d).^14,101^ Here we summarize the advances in the development of multispecific antibodies (mainly BsAbs) used for cancer treatment.

### The formats of multispecific antibodies

**Fig. 4:**
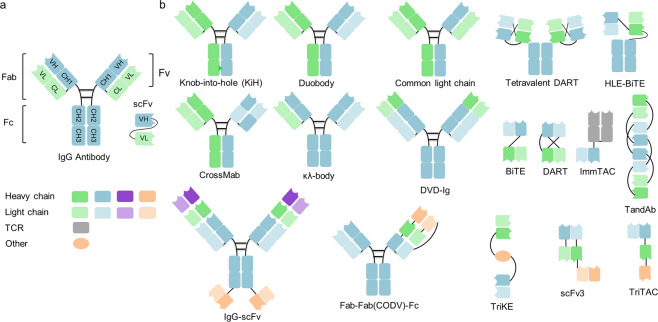
Schematic overview of the IgG antibody structure and representative multispecific antibody formats at clinical stages. **a** The classical IgG antibody structure that consists of Fab and Fc regions. The single chain variable fragment (scFv) is a combination of the variable region of heavy chain (VH) and variable region of light chain (VL) domains. **b** Various multispecific antibody formats that are FDA-approved or under clinical studies. They are classified into the following categories: IgG-like constructs (with Fc region), non-IgG-like fragments (without Fc region). ScFv single-chain variable fragment, DVD-Ig dual-variable-domain immunoglobulin, BiTE bispecific T-cell engager, HLE-BiTE half-life-extended BiTE, TandAb tandem diabody, DART dual-affinity retargeting molecule, CODV cross-over dual variable region, TriKE trispecific NK-cell engager, TriTAC trispecific T-cell activation constructs

In the 1960s, the original concept of mixing univalent antibody fragments to generate multispecific antibodies was first described by Nisonoff and colleagues.^102^ Initially, BsAbs were generated by chemical conjugation of two antibody fragments and then by somatic cell fusion of two different hybridoma cell lines. The development of recombinant DNA technology and antibody engineering technology made it possible to assemble different antibody domains into various formats of multispecific antibodies with a desired orientation and stability. In summary, the formats of multispecific antibodies can be classified into two major categories: IgG-like antibody formats (with an Fc domain) and non-IgG-like antibody formats (without an Fc domain) (Fig. 4). The full-length IgG-like multispecific antibody contains an Fc domain that can bind with the neonatal Fc receptor FcRn, showing better pharmacokinetic characteristics than antibody fragments and exhibiting multiple antitumor mechanisms.^103^ Novel strategies such as Knobs-into-holes and CrossMab enable the correct pairing of different Ig heavy and light chains (Fig. 4). Compared with full-length IgG-like multispecific antibodies, non-IgG-like multispecific antibodies lack the Fc domain and thus have a lower molecular weight, which enables greater penetration efficiency in solid tumors. As a result, antibody formats without an Fc or albumin-binding domain have a relatively short serum half-life owing to rapid renal clearance. Non-IgG-like multispecific antibodies without an Fc domain can potentially be used to prevent nonspecific activation of the innate immune system as well as Fc-mediated ADCC or CDC, which is crucial for reducing immune cell engagement-related side effects.^103^ The well-known multispecific antibody formats include dual-variable-domain Ig, tetravalent IgG-like antibodies, multispecific scFvs, IgG-scFv fusion proteins, multispecific Fabs, tandem VHH domains, and others (Fig. 4).

### Mechanisms of action of different multispecific antibodies for cancer treatment

Multispecific antibodies are engineered to target distinct antigen epitopes simultaneously and thus may exhibit synergistic therapeutic efficacy. The modes of action of multispecific antibodies include but are not limited to redirecting the human immune system to fight cancer, regulating a receptor signaling pathway, combinational targeting of different tumor antigens to increase tumor selectivity or mitigate antigen loss-related relapse. With suitable target combinations and optimal format design, multispecific antibodies may achieve the desired therapeutic outcome and are feasible for large-scale production. In the near future, an extensive pipeline of multispecific antibodies may provide valuable candidates to meet the needs for clinical cancer treatment.

### Targeting CD3 to engage T cells

Recruitable effector cells play critical roles in cancer immunotherapy. The early therapeutics BsAbs were T cell engagers, and their effects were first demonstrated in the mid-1980s.^104,105^ BiTEs are capable of binding CD3ε within the TCR complex and a selected tumor-associated antigen (TAA) on tumor cells simultaneously. Then, cytotoxic T cells are redirected to specifically kill the recognized tumor cells via T cell engagers.^15,106^ Catumaxomab (Removab^®^) was the first EMA-approved T cell-engaging BsAb for the treatment of malignant ascites; it was approved initially in 2009 and is currently used worldwide. As a trifunctional Triomab^®^ family member, catumaxomab consists of an anti-CD3 rat IgG2b half-antibody and an anti-epithelial cell adhesion molecule (anti-EpCAM) mouse IgG2a isotype, with one arm binding to a T cell and the other arm binding to a tumor cell.^107^ Additionally, the binding to Fcγ receptors on accessory cells (e.g., macrophages, monocytes, NK cells, and dendritic cells) via the functional Fc region results in highly concerted antitumor immune responses.^108^ The efficacy and safety of catumaxomab were demonstrated in a pivotal phase II/III study, and these results were corroborated in phase I/II studies.^107,109^ However, catumaxomab was withdrawn from the market in 2017 for commercial reasons. Currently, another clinical trial of catumaxomab is being conducted in China for treating patients with recurrent or metastatic gastric cancer with peritoneal metastasis (NCT04222114, ClinicalTrials.gov).

Another advanced T cell-engaging BsAb format at the forefront of cancer clinical trials is BiTE^®^ (Micromet, acquired by Amgen, Inc. in 2012). Blinatumomab (Blincyto; Amgen, Inc.) is composed of two scFvs targeting CD3ε on T cells and the B-lymphocyte antigen CD19. The striking clinical phase II trial results revealed a CR rate of 43% (81/189) in adult patients with R/R B-ALL after the first two cycles of continuous intravenous infusion of blinatumomab treatment^110^ (MT103–211, NCT01466179), which formed the basis for the accelerated approval granted by FDA in 2014 for the treatment of Philadelphia-chromosome negative (Ph−) R/R B-ALL in adults.^16^ Based on the encouraging clinical results of the TOWER trial (NCT02013167),^106,111^ Alcantara trial (NCT02000427),^112^ MT103–205 trial (NCT01471782)^113^ and BLAST trial (NCT01207388),^114^ the FDA approved the expansion of clinical indications for blinatumomab to patients with Philadelphia chromosome‐positive R/R B‐ALL and pediatric patients with R/R ALL or MRD-positive ALL.^115^

Recently, a chemotherapy-free first-line treatment strategy with blinatumomab and dasatinib (an ABL tyrosine kinase inhibitor (TKI)) showed high rates of molecular response and OS in 63 patients with Ph-positive ALL. Complete remission was observed in 98% of the patients, and at a median follow-up of 18 months, the OS was 95%, and the disease-free survival was 88%.^116^ Ongoing trials are evaluating the efficacy of blinatumomab administered in combination with chemotherapy, targeted therapies, or other immunotherapies, aiming to increase the OS rate and reduce side effects.^116–118^

The impressive clinical data of catumaxomab and blinatumomab sparked interest in the BsAb field and prompted researchers to develop a diverse variety of BsAb formats, leading to explosive growth in clinical trials. CD3-targeted BsAbs are rapidly becoming the most revolutionary strategic modalities in cancer immunotherapy, accounting for more than 50% of global BsAbs used in various clinical stages of cancer. Compared with IgG-like BsAbs, BiTEs show greater penetration into tumors owing to their small molecular weight (~55 kDa) and are relatively less immunogenic due to the lack of Fc domain-mediated effector toxicities.^15^ Researchers from Amgen have designed a series of BiTE^®^-based antibodies (anti-CD3 × tumor cell antigen) to validate their efficacy for use in cancer treatment; the drugs tested included AMG 330 (anti-CD3/CD33), AMG 427 (anti-CD3/FLT3, a half-life-extended BiTE® molecule in HLE-BiTE format, also known as a BiTE-Fc), AMG 673 (anti-CD3/CD33 in HLE-BiTE format), and AMG 701 (anti-CD3/BCMA in HLE-BiTE format) for use against hematological malignancies and AMG 160 (anti-CD3/a prostate-specific membrane antigen (PSMA) in HLE-BiTE format), AMG 596 (anti-CD3/epidermal growth factor receptor (EGFR) vIII), and AMG 757 (anti-CD3/DLL-3 in HLE-BiTE format) for use against solid malignancies; most of these drugs are still in the early stages of clinical development.

The recent spike of interest in antibody-mediated immune cell engagement for cancer treatment has driven many multifunctional antibodies into clinical studies. Approximately 30% of T cell-engaging BsAbs are in clinical trials for treating hematological malignancies, and the specific targets are mainly well-known TAAs, such as CD19, CD20, CD33, CD38, CD123, and BCMA.^110,119–123^ The application of T cell engaging BsAbs in solid tumors faces more-challenging hurdles, such as poor T cell infiltration, a complex immunosuppressive TME, and the higher likelihood of on-target off-tumor toxicities, raising concerns for the safety and efficacy of T cell engaging BsAbs. T cell-engaging BsAbs used for solid tumor treatment have been explored by targeting established tumor antigens, such as HER2, EpCAM, carcinoembryonic antigen (CEA), and PSMA.^124–127^ Many preclinical studies have demonstrated that T cell-engaging BsAbs can induce sufficient activation of T cells and subsequent killing of tumor cells in various mouse xenograft tumor models.^128–130^ To date, the most advanced T cell-engaging BsAbs that have entered clinical trials for investigation in patients are in phase I trials.^103^ In addition to the incorporation of extracellular antigens, some T cell-engaging BsAbs have incorporated TCR variable regions to recognize peptide/MHC I complexes that consist of MHC-I molecules and short peptides (8–9 aa) derived from intracellular proteins, and their binding is peptide-specific and MHC-restricted. Bispecific ImmTAC molecules (Immunocore, Inc.), fusion proteins composed of an affinity-enhanced TCR and an anti-CD3 scFv antibody, have provided potent and highly specific access to the vast landscape of intracellular targets via TCR-based tumor targeting. An exemplary ImmTAC molecule, tebentafusp (IMCgp100), which targets a gp100 peptide (a melanocyte differentiation antigen) presented by HLA-A02 on tumor cells, has shown promising clinical outcomes as a monotherapy agent for patients with metastatic uveal melanoma.^131^ The FDA has given a breakthrough therapy designation to tebentafusp (IMCgp100) for HLA-A2-positive, inoperable, or advanced uveal melanoma based on interim data obtained from the IMCgp100–202 Phase III trial (NCT03070392), which demonstrated the superiority of tebentafusp for extending survival in adults with newly diagnosed metastatic uveal melanoma. Tebentafusp has also been given a fast-track designation and orphan drug designation for use in uveal melanoma by the FDA and a Promising Innovative Medicine designation under the UK Early Access to Medicines Scheme for metastatic uveal melanoma. Currently, an IMCgp100–202 phase III clinical trial has met the predetermined criteria for statistical significance of the primary OS endpoint in tebentafusp treatment for patients with previously untreated metastatic uveal melanoma.

### Targeting T cell costimulatory receptors

Costimulatory receptors on T cells, such as 4–1BB (CD137) and OX40 (CD134), can be leveraged for cancer immunotherapy. To reduce hepatotoxicity after systemic T cell costimulation via mAbs (e.g., urelumab), anti-TAA antibodies were fused to agonistic antibodies recognizing costimulatory receptors for tumor localization, TAA-dependent clustering, and activation of costimulatory receptors, such as the HER2/4–1BB BsAb.^132^ Several costimulatory receptors, including CD28 and members of the TNF receptor (TNFR) superfamily, such as 4–1BB and OX40, are promising targets for T cell-costimulation-related cancer immunotherapy.^133,134^ For instance, the 4–1BB/HER2 bispecific molecule PRS-343 induced T cell costimulation by HER2-dependent 4–1BB clustering and activation, increasing the infiltration and activation of lymphocytes in tumors and thus significantly suppressing tumor growth in HER2+ mouse xenograft tumor models.^132^ A phase I clinical trial of the first-in-class 4–1BB/HER2 bispecific molecule PRS-343 was initiated to evaluate its in vivo antitumor efficacy. Recently, Wu et al. described the application of a novel trispecific antibody format, termed a crossover dual variable (CODV) format, to simultaneously target CD38, CD3, and CD28 for treating multiple CD38-positive hematological malignancies, such as MM, AML, and chronic lymphocytic leukemia.^135^ Dual engagement of CD3 and CD28, representing the first and second signals required for optimal T cell proliferation, respectively, afforded efficient T cell activation and costimulation and boosted sustained T cell proliferation to kill CD38-positive MM cells in vitro and in vivo.^135^

### Targeting CD16 to engage NK cells

NK cells have been identified as playing indispensable roles in the innate immune system, and growing evidence has demonstrated their ability to reduce tumor load and exert tumor immunosurveillance.^136^ CD16 (FcγRIII), mainly expressed on mature NK cells, is a low-affinity receptor that binds with the Fc domain of IgG antibodies to mediate ADCC. Bispecific and trispecific killer T cell engagers (BiKEs and TriKEs) are capable of exclusively triggering ADCC by crosslinking the CD16 receptor on NK cells together with antigen targets on cancer cells.^137^ AFM13 employs a tetravalent bispecific CD30/CD16A tandem diabody (TandAb®) with two binding sites for the CD16A isoform on NK cells and two binding sites for the CD30 antigen on lymphoma cells.^138,139^ Cytotoxicity assays demonstrated that AFM13 exhibited higher efficacy and potency with respect to the diabody and anti-CD30 IgG antibodies and showed inhibited lysis of CD30+ target cells.^139^ Early clinical studies suggested that AFM13 represents a well-tolerated, potent NK cell-engaging immunotherapy for treating CD30+ malignancies.^140,141^ A phase I dose-escalation study with patients with R/R HL revealed that the overall disease control rate of AFM13 was 61.5% in 26 evaluable patients, and the partial remission rate reached 11.5%.^140^ Another phase Ib study of AFM13 administered in combination with pembrolizumab in patients with R/R HL recently showed an 83% overall response rate for all recipients, and it showed good tolerability.^141^ In addition, a novel anti-CD16 × IL-15 × CD33 TriKE (GTB-3550/OXS-35504) construct designed by Vallera et al. incorporated an IL-15 cross-linker within scFvs recognizing CD16 on NK cells and CD33 on myeloid tumor cells.^142,143^ In vitro and in vivo studies revealed that GTB-3550 engaged NK cells to induce ADCC and eradicate CD33+ tumor cells efficiently, indicating the profound impact of IL-15 on maintaining the survival and proliferation of NK cells.^142,143^ A phase I/II clinical trial (ClinicalTrials.gov NCT03214666) for the treatment of CD33-positive leukemias such as AML, myelodysplastic syndrome, and other CD33+ hematopoietic malignancies is ongoing. The successful application of NK cell engagers in clinical stages of cancer strongly suggests that BsAb-mediated NK cell engagement is an alternative and viable strategy for leveraging immune responses to cancer. Notably, a higher proportion of NK cell infiltration within tumors has been demonstrated to be associated with better clinical outcomes in patients.^144^ Collectively, NK cell engagers hold promise in the near future as the most powerful agents for treating hematological malignancies and solid tumors.

### Targeting checkpoint receptors on T cells

In an alternative strategy implemented following the improved clinical benefit observed in patients, the results from checkpoint blockade immunotherapy have shed light on manipulating the activation of immune cells in cancer treatment.^145,146^ Several studies have shown that the upregulated expression level of PD-L1 on advanced tumor cells inhibited the T cell response and facilitated cancer cell immune evasion, which limited the in vivo efficacy of cancer therapies.^147,148^ The utilization of therapeutic antibodies to target checkpoint molecules, including cytotoxic T lymphocyte antigen-4 (CTLA-4), PD-1, and PD-L1, unleashed pre-existing antitumor immune responses.^149^ Moreover, checkpoint blockades have been incorporated into BsAbs to achieve tumor-localized and TAA-dependent checkpoint blockage.^150,151^ For example, an anti-PD-1 × HER2 (human epidermal growth factor receptor 2) BsAb (IBI315, Innovent, Inc., China) was designed to bridge PD-1-expressing T cells to HER2-expressing tumor cells, block the PD-1/PD-L1 signaling pathway in a HER2-dependent manner, and inhibit activation of the HER2 signaling pathway.^152^ As a result, IBI315 combines targeted therapy with immunotherapy, thereby potentially enhancing antitumor activity via multiple mechanisms of action. Currently, a phase Ia/Ib clinical study is evaluating the safety, tolerability, and efficacy of IBI315 in patients with HER2-expressing advanced solid tumors (NCT04162327). Similarly, an anti-PD-1 × EGFR IgG scaffold-based BsAb has exhibited potent antitumor efficacy and enhanced T cell-based antitumor immunity through PD-L1 blockade in both in vitro and in vivo models.^151^ Simultaneous blockade of two immune checkpoints with synergic mechanisms of action has been used to maximize checkpoint blockade in the TME.^153,154^ A clinical trial showed that five-year outcomes of applying nivolumab (anti-PD-1) plus ipilimumab (anti-CTLA-4) to treat patients with advanced melanoma led to sustained long-term OS compared with treatment with ipilimumab alone,^155^ providing a rationale for concurrently targeting two immune checkpoints. Anti-PD-1 × CTLA-4 BsAbs, including AK104,^156^ MEDI5752,^157^ and MGD019,^158^ are expected to synergistically inhibit checkpoint signaling pathways in PD-1/CTLA-4 double-positive tumor-infiltrated lymphocytes (TILs) while enhancing PD-1 degradation and CTLA-4 inhibition in the TME.^157^

Moreover, to reverse acquired T cell-anergy-mediated resistance, the idea of targeting novel inhibitory receptors expressed on TILs, such as LAG-3 (lymphocyte-activation gene-3) and TIM-3 (T cell immunoglobulin and mucin-domain-containing molecule 3) with or without PD-1 and/or PD-L1, is also being explored in early clinical trials^159^ (NCT03219268, NCT03708328, and NCT03440437). A T cell-costimulation pathway can be combined with an immune checkpoint axis blockade to achieve sustained antitumor immune responses against advanced cancer.^160,161^ ICOS (inducible T cell costimulator, also known as CD278) and TNFR superfamily members such as 4–1BB and OX40 are promising targets of therapeutic BsAbs. The combinations of immune checkpoint blockade and costimulatory receptor activation, such as PD-L1 × 4–1BB (MCLA-145, FS222), CTLA-4 × OX40 (ATOR 1015), and PD-1 × ICOS (XmAb23104), are under preclinical and clinical investigation.^162–165^ To prevent potential toxic side effects due to unspecific overactivation, a monovalent trispecific antibody NM21–1480 comprising three scFvs (αPD-L1, α4–1BB and αHSA) fused in a single chain was designed to restrict 4–1BB signaling upon PD-L1 blockage.^166^ A similar immunomodulatory design strategy was used to construct GNC-038, a tetraspecific IgG-scFv-conjugated antibody (αCD19/CD3/4–1BB/PD-L1), now in phase I clinical trial (NCT04606433).

Recently, Cotton et al. showed that the fully recombinant BsAb AC-1, an antibody-based proteolysis-targeting chimera (PROTAC) termed AbTAC, simultaneously bound PD-L1 and the membrane-bound E3 ligase RNF43 to degrade PD-L1 by recruiting RNF43.^167^ This AbTAC efficiently recruited membrane-bound E3 ligase to degrade cell-surface PD-L1 in different cell lines with high PD-L1 expression levels (MDA-MB-231, HCC827, and T24 cells) via lysosomal degradation.^167^ AbTACs represent a new archetype of BsAbs for use in the targeted degradation of cell-surface proteins, further expanding the range of BsAb applications.

### Targeting CD47 to enhance macrophage-mediated phagocytosis

CD47 is widely expressed on both healthy and cancer cells and transmits a “do not eat me” signal upon interaction with the signal-regulatory protein alpha (SIRPα) receptor on myeloid cells, including monocytes, macrophages, neutrophils, and a subset of dendritic cells.^168^ Blockade of the CD47-SIRPα axis by anti-CD47 mAbs augmented antitumor activity by unleashing macrophage-mediated phagocytosis; however, it also increased the possibility of damaging normal CD47-positive cells, such as erythrocytes.^169,170^ CD47-based BsAbs represent s viable strategy for protecting red blood cells while maximizing the potency of CD47 blockade. The introduction of a tumor-specific targeting arm into CD47-based BsAbs may potentially restrict CD47-SIRPα blockade to the tumor site. TG-1801 (also known as NI-1701, TG Therapeutics, Inc.) is a 1:1 IgG1 bispecific κλ antibody (CD19 × CD47) that combines malignant B cell targeting and selective CD47 blockade.^171,172^ Accordingly, TG-1801 specifically targeted double-positive tumor cells and showed antitumor efficacy through ADCP in vitro and in vivo.^171^ A phase I clinical trial for evaluating the safety and optimal dosage of TG-1801 (NCT03804996) is ongoing. Another anti-CD47-based BsAb, N1801 (TG Therapeutics, Inc.) was designed to target CD47 and mesothelin (MSLN), an antigen overexpressed in multiple solid tumors, and was demonstrated to efficiently kill MSLN+ tumor cells without generating an adverse hematological profile in a nonhuman primate study (AACR Annual Meeting 2019).^173,174^ The synergistic activation of innate and adaptive immune responses is also promising for tumor immunotherapy because it leads to the modulated activity of two highly potent effector cells, NK cells, and T cells. For example, the dual blockage of the PD-1/PD-L1 axis and CD47-SIRPα axis has been incorporated into single BsAbs, namely, IBI322 (PD-L1 × CD47) and HX009 (PD-1 × CD47), to simultaneously potentiate the local adaptive and innate immune responses against tumor cells and is being evaluated in phase I trials (NCT04338659 and NCT04097769 for IBI322 and HX009, respectively).^175^

### Targeting receptor signaling pathways in tumor cells

Multispecific antibodies that recognize different receptors involved in signaling crosstalk can be utilized to obviate bypass signal transmission in cancer development.^176–178^ Receptor tyrosine kinases, such as EGFR, HER2, HER3, and MET, are enzyme-linked transmembrane receptors that consist of an extracellular ligand-binding domain, a transmembrane domain, and an intracellular protein–tyrosine kinase domain.^179^ Multiple studies have shown that phosphorylation and activation of c-Met signaling contribute to the resistance of EGFR TKIs in patients with non-small cell lung cancer (NSCLC).^180^ Recently, it was reported that the combination of osimertinib (EGFR TKI) plus savolitinib (MET TKI) showed an acceptable safety profile and encouraging antitumor efficacy in patients with advanced NSCLC.^181^ Therefore, dual inhibition of aberrant cell survival or proliferation-related signaling pathways has become a promising strategic focus for treating patients with MET-driven resistance to EGFR TKIs. Amivantamab/JNJ-61186372 is a dual-targeting EGFR × MET BsAb generated through controlled Fab arm exchange and can inhibit EGFR and MET signaling pathways while triggering Fc-mediated effector interactions.^177^ In various tumor models, amivantamab efficiently induced the downmodulation of EGFR and MET expression and exhibited immune-directed antitumor activity, translating clinically into robust antitumor responses in two exon 20ins patients.^182^ Notably, on May 21, 2021, the FDA granted accelerated approval to JNJ-61186372 (Rybrevant, Janssen Biotech, Inc.) for the treatment of adult patients with locally advanced or metastatic NSCLC with EGFR exon 20 insertion (exon 20ins) mutations. Another example is MCLA 128, an IgG1 BsAb specifically binding HER2 and HER3, which potently inhibits heregulin-driven signaling of HER2/HER3 and downstream PI3K/Akt signaling to suppress tumor cell survival and proliferation.^183^ In addition to those directed to breast cancer, clinical studies on MCLA 128 are ongoing in patients with metastatic pancreatic ductal adenocarcinoma or NSCLC that harbor fusion neuregulin 1 (NRG1) genes.

In addition to targeting extracellular membrane receptors, multifunctional antibodies can be employed to neutralize active mediators in the TME. Importantly, the inhibition of angiogenesis in tumor tissue, also known as tumor starvation, has been shown to be an effective way to deprive solid tumor cells of oxygen and nutrients needed for growth and metastasis.^184,185^ RO5520985/Vanucizumab, a BsAb in crossMab format, acts as a dual-target inhibitor of angiogenin-2 and vascular endothelial growth factor-A (VEGF-A).^186^ Preclinical studies have demonstrated that RO5520985 can induce potent antiangiogenic and tumor growth inhibition and antimetastatic efficacy in patient-derived tumor xenograft models.^186^ A first-in-human phase I study was performed to evaluate the safety and antitumor activity of RO5520985 as a monotherapy, and the results showed no disease progression in ten patients for ≥6 months and the induced toxicity was generally manageable.^187^ However, the McCAVE trial was terminated because the Vanucizumab/mFOLFOX-6 treatment did not improve the PFS of patients with previously untreated metastatic colorectal carcinoma.^188^ Thorough mechanistic studies and well-designed clinical trials with multispecific antibodies are needed to determine the principles underlying multiple-signaling pathway modulation in the TME.

### Combinational targeting of TAAs

Tumor-specific targeting is important for therapeutic antibodies to minimize on-target off-tumor toxicity because TAAs are also expressed in noncancerous cells. To increase the specificity of tumor targeting, one appealing approach is to incorporate different antigen-binding modules into one multispecific antibody modality. The density, specificity, and stability of a target antigen on the surface of the tumor cell affect the duration of efficacious antitumor treatment with multispecific-antibody-engaged effector cells. Many receptor targets are subject to enzymatic cleavage that releases the soluble extracellular domain, e.g., CEA, HER2, CD19, and BCMA, thereby leading to variable and heterogeneous target receptor expression on tumor cells and even antigen escape.^189^ Multispecific antibodies with different targeting abilities are viable agents for limiting antigen escape and increasing the specificity of tumor targeting. For example, a dual-target single‐chain Fv antibody (CD123 × CD33) displayed more effective antitumor responses than a monotarget antibody in AML.^190^ Another example is DT2219ARL, a bispecific single-chain fusion protein that recognizes CD19 and CD22 and is fused with a diphtheria toxin (DT390) variant.^191^ Although CD19 is a validated target in B-ALL treatment, antigen loss-mediated relapse is a major limitation of potent CD19-targeted immunotherapies.^192^ However, CAR-T cells targeting CD22, which is also highly expressed on B cell malignant cells and are retained after CD19 loss, were found to be effective in killing CD22-positive B-ALL cells.^193^ A phase I trial of DT2219ARL for refractory B cell malignancy treatment demonstrated its safety, and the optimal dosage was determined.^194^ Complete remission or partial response (PR) in a 1-year timeframe was observed in 2 of 18 enrolled in a phase I/II trial (NCT02370160).

The strategy of combining targeting tumor antigens has also been applied to chimeric antigen receptor (CAR) T cell therapy and is a promising immunotherapy for several malignancies, such as lymphomas, leukemias, and MM.^195–197^ T cells obtained from a patient are engineered to express CARs in vitro. Recent advances have enabled the development of CAR T cells that target multiple antigens on the tumor cell surface, such as bispecific CARs and tandem CARs (Tan‑CARs), to overcome target antigen escape in patients with relapsed and refractory malignancies.^197–199^ A CAR is composed of an extracellular antigen-binding domain derived from a mAb (e.g., scFv, Fab, or VHH domain), a transmembrane domain, and an intracellular signaling domain.^200,201^ Bispecific CARs, with two distinct antibodies or antigen-binding domains (e.g., tandem VHH domains or scFvs) on one CAR T cell, can recognize different antigens or epitopes in one antigen during the targeting of tumor cells.^202^ For example, bispecific anti-CD20/CD19 (LV20.19) CAR T cells have shown low toxicity and high efficacy in the treatment of relapsed, refractory B cell malignancy (NCT03019055).^203,204^ Similarly, LCAR-B38M, a dual BCMA epitope-binding CAR T cell, demonstrated clinical efficacy and a manageable safety profile in patients with relapsed/refractory (R/R) MM.^205^

In a different strategy of cancer cell dual targeting, multispecific antibodies can be designed to recognize antigens on different tumor cells for the purpose of tumor-restricted activation with limited toxicity induction. For example, RG7386/RO6874813, a symmetric tetravalent 2:2 CrossMab BsAb that possesses a high affinity for fibroblast activation protein (FAP) on tumor stromal fibroblasts and low affinity for death receptor 5 (DR5), potently triggered death receptor-mediated apoptosis of tumor cells while sparing normal cells.^206^ This anti-FAP×DR5 BsAb demonstrated superior antitumor efficacy against FAP-expressing malignant cells in vitro as well as in patient-derived xenograft mouse models compared to that of conventional DR5 antibodies.^206^ A list of FDA-approved and clinical-stage multispecific antibodies is presented in Table 3.

**Table 3:** The FDA-approved and clinical-stage multi-specific antibodies

Name	Target	Format	Indication(s)	Status	ClinicalTrials.gov identifier	Reference
Blinatumomab/Blincyto/MT103/MEDI-538/AMG103	CD3, CD19	BiTE	Hematological malignancies	Marketed	NCT01466179 NCT02013167	^110,111^
AFM11	CD3, CD19	Tandem diabody (TandAb)	Relapsed B cell non-Hodgkin lymphoma	Terminated	NCT02848911	^342^
AMG562	CD3, CD19	HLE-BiTE	Hematological malignancies	Phase I	NCT03571828	^343^
REGN1979	CD3, CD20	Common light chain	Non-Hodgkin lymphoma	Phase II	NCT03888105	^120^
Glofitamab/RO7082859	CD3, CD20	Fab-Fc (IgG1) × Fab-Fab-Fc (IgG1), CrossMab	Non-Hodgkin lymphoma	Phase I	NCT03075696	^344^
Plamotamab/XmAb13676	CD3, CD20	Fab-scFv-Fc	Non-Hodgkin lymphoma	Phase I	NCT02924402	^345^
Mosunetuzumab/RG7828/RO7030816	CD3, CD20	Knob-into-hole (KiH)	B cell lymphoma	Phase I	NCT04313608	^346^
GEN3013	CD3, CD20	DuoBody	B cell lymphoma	Phase I	NCT03625037	^119^
AMG673	CD3, CD33	HLE-BiTE	Relapsed/refractory acute myeloid leukemia	Phase I	NCT03224819	^122^
AMV-564	CD3, CD33	Tandem diabody (TandAb)	Acute myeloid leukemia	Phase I completed	NCT03144245	^347^
ISB 1342	CD3, CD38	Fab-Fc (IgG1) × scFv-Fc (IgG1)	Multiple myeloma	Phase I	NCT03309111	^348^
JNJ-63709178	CD3, CD123	DuoBody	Relapsed or refractory acute myeloid leukemia (AML)	Phase I completed	NCT02715011	^349^
SAR440234	CD3, CD123	CODV-Fab-TL1	Leukemia	Terminated	NCT03594955	^350^
Vibecotamab/Xmab14045	CD3, CD123	Fab-scFv-Fc	Hematologic malignancies	Terminated	NCT02730312	^121^
AMG420/BI 836909	CD3, BCMA	BiTE	Relapsed and/or refractory multiple myeloma	Phase I	NCT03836053	^351^
CC-93269/EM801	CD3, BCMA	CrossMab, KiH	Relapsed and/or refractory multiple myeloma	Phase I	NCT03486067	^352^
Teclistamab/JNJ-64007957	CD3, BCMA	Duobody	Hematological malignancies	Phase II	NCT04557098	^123^
PF-06863135	CD3, BCMA	DuoBody	Multiple myeloma	Phase II	NCT04649359	^353^
REGN5458	CD3, BCMA	Fab-Fc-Fab	Multiple myeloma	Phase I/II	NCT03761108	^354^
Catumaxomab/removab	CD3, EpCAM	TrioMab	Malignant ascites	Withdrawn from the market	/	^109^
Marketed	CD3, gp100	ImmTAC	Uveal melanoma	Phase III	NCT03070392	^131^
RG6194/BTRC4017A	CD3, HER2	Undisclosed	Solid tumors	Phase I	NCT03448042	^355^
M802	CD3, HER2	YBODY	HER2-positive solid tumors	Phase I	NCT04501770	^356^
GBR1302	CD3, HER2	Fab-scFv-Fc	Breast cancer	Terminated	NCT03983395	^357^
Cibisatamab/RG7802/RO6958688	CD3, CEA	2:1 CrossMab	Colorectal cancer	Phase I	NCT03866239	^358^
AMG211	CD3, CEA	BiTE	Advanced gastrointestinal cancer	Terminated	NCT02291614	^359^
AMG160	CD3, PSMA	HLE-BiTE	Prostate cancer	Phase I	NCT03792841	^360^
MOR209/ES414	CD3, PSMA	scFv-Fc (IgG1)-scFv	Prostate cancer	Phase I completed (discontinued)	NCT02262910	^361^
Pasotuxizumab/BAY2010112	CD3, PSMA	BiTE	Prostate cancer	Phase I completed	NCT01723475	^129^
REGN5678	CD28, PSMA	Fab-Fc (IgG4)-Fab	Prostate cancer	Phase I/II	NCT03972657	^362^
FS120	OX40/4–1BB	Tetravalent mAb^2^	Advanced malignancies	Phase I	NCT04648202	^363^
PRS-343	HER2/4–1BB	Anticalin-mAb	HER2-positive solid tumors	Phase I	NCT03330561	^132^
AFM13	CD16A, CD30	Tandem diabody (TandAb)	Lymphoma	Phase I/II	NCT03192202, NCT04101331	^140,141^
AFM24	CD16A, EGFR	Tandem diabody (TandAb)	Advanced solid tumor	Phase I	NCT04259450	^364^
GTB-3550, OXS-35504	CD16, CD33, IL-15	Tri-specific killer engager (TriKE)	Hematological malignancies	Phase I/II	NCT03214666	^142^
MEDI5752	PD-1, CTLA-4	Common light chain	Advanced renal cell carcinoma, selected advanced solid tumors	Phase I	NCT04522323	^157^
AK104	PD-1, CTLA-4	Undisclosed	Advanced solid tumors	Phase I/II	NCT04172454	^156^
XmAb20717	PD-1, CTLA-4	Fab-scFv-Fc	Advanced solid tumors	Phase I	NCT03517488	^365^
MGD019	PD-1, CTLA-4	DART-Fc	Advanced solid tumors	Phase I	NCT03761017	^158^
MGD013	PD-1, LAG-3	Tetravalent DART	Solid and hematological malignancies	Phase I	NCT03219268	^366^
RO7121661, RG7769	PD-1, TIM3	CrossMab, KiH	Solid tumors	Phase I	NCT03708328	^367^
KN046	PD-L1, CTLA-4	Common light chain	Advanced solid tumors (triple-negative breast cancer, squamous non-small cell lung cancer, thymic carcinoma), lymphoma	Phase I	NCT03872791, NCT04474119, NCT04469725, NCT03733951	^368^
FS118	PD-L1, LAG-3	Tetravalent mAb^2^	Advanced malignancies	Phase I	NCT03440437	^159^
LY3415244	PD-L1, TIM3	Undisclosed	Solid tumor	Phase I terminated	NCT03752177	^369^
IBI318/LY3434172	PD-1, PD-L1	Undisclosed	Advanced malignancy	Phase I/II	NCT03875157	^370^
IBI315	PD-1, HER2	Undisclosed	Advanced solid tumor	Phase I	NCT04162327	^152^
AK112	PD-1, VEGF	Tetrabody	Advanced solid tumor malignancies	Phase I	NCT04047290	^150^
IBI319	PD-1, 4–1BB	Knob-into-hole	Advanced malignant tumors	Phase I	NCT04708210	^371^
FS222	PD-L1, 4–1BB	mAb^2^	Advanced cancer, metastatic cancer	Phase I	NCT04740424	^163^
MCLA-145	PD-L1, 4–1BB	Common light chain	Advanced or metastatic malignancies	Phase I	NCT03922204	^165^
ATOR 1015	CTLA4, OX40	mAb × Ligand	Solid tumors	Phase I completed	NCT03782467	^162^
XmAb23104	PD-1, ICOS	Xmab	Solid malignancies	Phase I	NCT03752398	^164^
TG-1801/NI-1701	CD47, CD19	κλ body	B cell lymphoma	Phase I	NCT03804996	^171^
IMM0306	CD47, CD20	Fab × Ligand-Fc (IgG1)	Non-Hodgkin lymphoma	Phase I	CTR20192612	^372^
IBI322	CD47, PD-L1	Undisclosed	Advanced malignancies	Phase I	NCT04338659, NCT04328831	^373^
HX009	CD47, PD-1	Undisclosed	Advanced solid tumor	Phase I	NCT04097769	^374^
JNJ-61186372/Amivantamab	EGFR, MET	Duobody	Non-small cell lung cancer	Marketed	NCT02609776	^375^
MCLA-158	EGFR, LGR5	Common light chain	Advanced solid tumors	Phase I completed	NCT03526835	^376^
MCLA-128/Zenocutuzumab	HER2, HER3	Common light chain	Breast cancer	Phase I/II	NCT03321981	^377^
KN026	HER2, HER2	Common light chain	HER2-positive solid tumors	Phase I	NCT04521179	^378^
MBS301	HER2, HER2	KiH	HER2-positive solid tumors	Phase I	NCT03842085	^379^
ZW25	HER2, HER2	Common light chain	HER2-positive solid tumors	Phase I	NCT02892123	^380^
ZW49	HER2, HER2 ADC	scFv-Fc (IgG1) × Fab-Fc (IgG1), conjugated to auristatin	HER2-positive solid tumors	Phase I	NCT03821233	^381^
MM-141	IGF-1R, HER3	scFv-IgG	Metastatic pancreatic cancer	Phase II completed	NCT02399137	^382^
BI 836880	ANG2, VEGF	Tandem V_HH_	Neoplasms	Phase I/II	NCT03972150, NCT03697304	^383^
RO5520985/Vanucizumab	ANG2, VEGF	CrossMab	Neoplasms	Phase II terminated	NCT02141295	^186^
ABT-165/Dilpacimab	DLL4, VEGF	Dual-variable-domain antibody (DVD-Ig)	Advanced solid tumors	Phase I	NCT01946074	^384^
OMP-305B83/Navicixizumab	DLL4, VEGF	Common light chain	Ovarian, peritoneal or fallopian tube cancer	Phase I completed	NCT03030287	^385^
RG7386/RO6874813	FAP, DR5	2:2 CrossMab	Solid tumor	Phase I completed	NCT02558140	^206^
OXS-1550/DT2219ARL	CD19, CD22	scFv-scFv toxin	Relapsed or refractory B-lineage leukemia or lymphoma	Phase I/II completed	NCT02370160	^191^

## Antibody–cytokine fusion proteins

Cytokines, such as ILs (e.g., IL-2), interferons (IFNs; e.g., IFN-γ), colony-stimulating factors (e.g., G-CSF), TNFs (e.g., TNF-α), and chemokines (e.g., CXCL3), are small proteins produced by immune cells or nonimmune cells and play vital roles in enhancing the efficacy of biological drugs. A complex network is formed by cytokines through their pleiotropic effects as well as complex autocrine and paracrine endocrine effects.^207^ At the same time, the high efficiency of cytokines drives the evolution of strict negative regulation mechanisms, which greatly weaken their biological functions.^207^ Moreover, dose-dependent side effects, unfavorable pharmacokinetics, poor drug tolerance, and high toxicity also limit their applications.^19^ With great progress in cancer research, the remarkable success of therapeutic antibodies in treating various cancer types has sparked interest in the development of novel immunotherapies administered alone or in combination.^22^ Fusion of cytokines to antibodies or antibody fragments may lead to more-specifically targeted TAAs, which may improve the efficacy, pharmacokinetics, and local concentration of cytokines and prevent systemic toxicity (Fig. 1d).^208^ A list of representative antibody–cytokine fusion proteins is presented in Table 4.

**Table 4:** Representative clinical-stage immunocytokines

Name	Cytokine	Target	Format	Indication(s)	ClinicalTrials.gov identifier	Status
L19IL2	IL2	Fibronectin ED-B	scFv-IL2	Solid tumors Renal cell carcinoma	NCT01058538	Phase I/II
L19TNFa	TNFα	Fibronectin ED-B	scFv-TNFα	Solid tumors Colorectal cancer	NCT01253837	Phase I/II
F16IL2	IL2	Tenascin C	scFv-IL2	Solid tumor Breast cancer Metastatic melanoma Non-small cell lung cancer	NCT01134250	Phase Ib/II
hu14.18-IL2	IL2	GD2	IgG1-IL2	Melanoma (skin) Neuroblastoma Sarcoma Unspecified childhood solid tumor	NCT00003750	Phase II
huKS-IL2 (EMD 273066)	IL2	EpCAM	IgG-IL2	Lung cancer Prostate cancer Ovarian cancer	NCT00132522	Phase I
DI-Leu16-IL2 (anti-CD20-IL2)	IL2	CD20	IgG-IL2	B cell non-Hodgkin lymphoma	NCT01874288	Phase I/II
NHS-IL12	IL12	EpCAM	IgG-IL12	Solid tumor Colon cancer Kaposi sarcoma	NCT04303117	Phase I
NHS-IL2-LT (EMD 521873)	IL2	DNA/histone complex	IgG-IL2	Lung cancer Non-small cell lung cancer	NCT00879866	Phase I
Anti-CEA-IL2v (cergutuzumab amunaleukin)	Variant of IL 2	Carcinoembryonic antigen (CEA)	IgG-IL2	Solid tumors	NCT02350673	Phase I/II

**Fig. 5:**
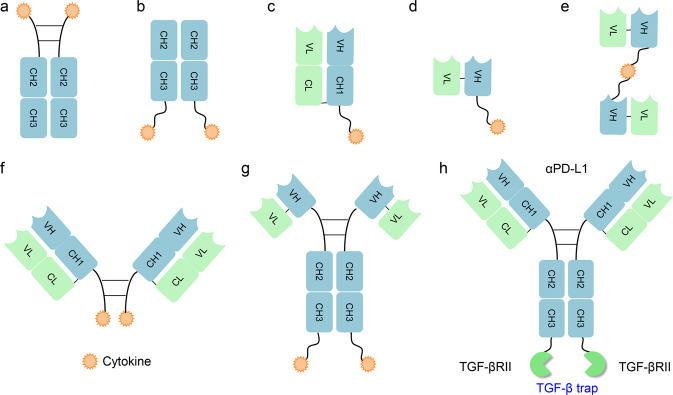
Different antibody or antibody fragment-cytokine fusion proteins. **a** Cytokine fused to the N-terminus of the Fc domain; **b** Cytokine fused to the C-terminus of the Fc domain; **c** Cytokine fused to the C-terminus of the Fab; **d** Cytokine fused to the C-terminus of the scFv; **e** ScFv-cytokine-scFv fusion protein; **f** F(ab')-cytokine fusion protein; **g** IgG format immunocytokine without CH1 and CL; **h** Representative IgG format immunocykine-Bintrafusp alfa (M7824). A bifunctional antibody fusion protein composed of anti-PD-L1 human IgG1 and human TGF-βRII extracellular domain as the TGF-β trap, though a flexible glycine-serine linker fused to CH3-C terminus of IgG. Bintrafusp alfa blocks PD1-PDL1 as well as TGFR2-TGFβ signaling pathways to relieve immune suppression and remove the immune inhibition

A variety of antibody–cytokine fusion proteins that can be classified into different categories based on their structures have been identified in recent decades (Fig. 5).^208,209^ An intact IgG, Fc, Fab or scFv can be fused with either monomers (e.g., IL-2 or IFN-α) or homomultimers of cytokines (e.g., IFN-γ or TNF).^207^ In addition, for some cytokines with two different polypeptide chains or heteromultimers (e.g., IL-12 and IL-27), different fusion strategies can be considered. Optimal combinations of antibodies or antibody fragments with cytokines may be acquired by considering various essential aspects (e.g., antibody type and fusion strategy) before applications are tested in clinical contexts. In principle, fusion with an IgG antibody or Fc domain conveys inherent advantages, such as binding to the neonatal Fc receptor (FcRn) and specific effector functions, to cytokines of interest to improve their pharmacokinetics, in vivo stability and in vivo efficacy. Furthermore, antibodies also provide a specific binding ability to target proteins to facilitate the efficient localization of cytokines to tumors in the form of immunocytokines. However, proinflammatory immunocytokines can trigger further cytokine production. In the clinic, the main side effects observed are hypotension, vomiting, flu-like symptoms, and nausea. Dose-dependent side effects and systemic toxicity can be reduced by reducing administration frequency.^22^ High-dose IL-2 (up to 800 million international units (IU)) administered for one week induces substantial toxicity,^210^ and therefore, IL-2-based immunocytokines are usually injected at much lower doses, such as 20–60 million IU, once a week for >6 months. It has been reported that the median half-life of an antibody–cytokine fusion protein, hu14.18-IL-2, which has been used for the treatment of refractory or recurrent neuroblastoma and melanoma, was only 3.1 h in clinical application.^211^ Therefore, the half-life of the fusion protein and its therapeutic effect were disappointing. Most likely, the accumulation of the fusion protein around a tumor might change the microenvironment, including densely packed activated immune cells, which may cause steric hindrance and block antigen presentation. In addition, the format and payload should be reconsidered during fusion protein development.^19^

### IL-2 fusion protein

IL-2 is mainly produced by CD4^+^ T cells^212^ and can stimulate the immune system pleiotropically, including activation of NK cells, cytotoxic T cells, and monocytes. Human IL-2 displays biological activity by binding to the human IL-2 receptor (IL-2R), which consists of α, β, and γ subunits. Different subunits perform different functions. IL-2Rα (also known as p55 or CD25) binds to IL-2 with low affinity; IL-2Rβ (also known as p75 or CD122) mainly binds to Janus kinase 1 (JAK1) and is essential to the active signal transducer and activator of transcription 5 (STAT5); IL-2Rγ (also known as p64 or CD132) is essential for proliferative signaling, but the γ subunit alone does not show affinity for IL-2.^213^ Based on the function of IL-2, the development of fusion proteins that selectively favor the activation of IL-2Rβγ, but not IL-2Rα, may specifically activate T cells or NK cells to kill tumor cells while preventing side effects. OMCP-mutIL-2 is composed of a mutated form of IL-2 with a poor affinity for IL-2Rα and a cowpox virus encoding the NKG2D-binding protein (OMCP).^214^ This fusion protein (OMCP-MUTIL-2) activates IL-2 signaling effectively only in NKG2D cells, such as NK cells, not in IL-2Rα-positive cells. OMCP-MUTIL-2 has shown superior antitumor efficacy in several mouse xenograft models without inducing severe toxicity.

Since 1984, high-dose IL-2 has been used to treat malignant melanoma patients with tumor regression.^215^ In 1992 and 1998, the FDA approved IL-2 for the treatment of metastatic renal cell carcinoma and metastatic melanoma, respectively. However, high-dose side effects and vascular leakage syndrome, as well as a short serum half-life, were identified as major obstacles that hampered the therapeutic effect of IL-2.^216^ To address these issues, various antibody-IL-2 fusion proteins, such as IL-2 fused with an intact IgG, scFv, or a Fc domain, have been generated to produce higher local concentrations, reduce the effective dose and prolong the half-life of IL-2.

The immunocytokine NHS-IL-2-LT2 (EMD 521873), whose antibody moiety targets the DNA-histone complex in necrotic tumor cells, showed low toxicity when administered as a single agent in vivo.^217^ An IgG-IL-2 fusion protein enhanced the pharmacokinetic properties of IL-2 and increased its half-life from 7.8 to 11 h, similar to the effect of an IgG antibody (~150 kDa).^218^ A new IL-2 mutant (FSD13), with high efficacy and low toxicity, was identified by site-directed mutagenesis at IL-2 interaction sites and was obtained by replacing the proline residue at the 65th position with a lysine residue.^219^ The IL-2 mutant FSD13 was more potent than wild-type human IL-2 in activating CD4^+^ T cells, CD8^+^ T cells, and NK cells but was threefold weaker in inducing the conversion of CD4^+^ T cells into T_reg_ cells.^219^ In addition, FSD13 significantly inhibited the growth and metastasis of melanoma without causing severe adverse side effects in the liver or lungs of a mouse xenograft model.^219^ The preclinical antitumor activity of the IL-2 mutant FSD13 was further investigated in combination with immune checkpoint blockers.^219^

### IL-12 fusion protein

IL-12, composed of two subunits, p40 and p35, is a proinflammatory cytokine produced by antigen-presenting cells. IL-12 can induce the proliferation of NK and T cells and the production of IFN-γ, which results in the activation of T cells and the differentiation of Th1 cells.^220^ The co-inoculation of tumor cells with IL-12-secreting fibroblasts markedly inhibited tumor growth and neovascularization surrounding tumors in immunodeficient mice, suggesting a potent antitumor effect of IL-12.^221^ Furthermore, IL-12 showed cytotoxic potency against MHC-negative tumor cells, which was likely a result of the macrophage-mediated production of nitric oxide and activation of NK cells.^222,223^ IL-2 upregulated the expression of IFN-γ and IFN-γ-inducible protein 10 (IP-10) in endothelial cells. IL-12 was found to play a vital role in creating an inflammatory microenvironment during cancer treatment.^224^ However, in clinical trials, the local concentration of IL-12 at tumor sites was much lower than the overall administration dosage, inducing unfavorable immune responses and severe systemic toxicity.^223,225^

Murine IL-12 (mIL-12) was fused to scFv L19 of the human antibody L19, which recognizes the extra domain B (ED-B) domain of human fibronectin, an angiogenesis marker secreted by endothelial cells and tumor cells, to generate the protein L19-(mIL-12). The antitumor effect of fusion protein L19-(mIL-12) was enhanced in a syngeneic murine lung metastasis tumor model.^226^ A heterodimeric fusion protein including two scFv moieties, with one scFv fused to the N-terminus of the p35 subunit and another scFv fused to the C-terminus of the p40 subunit, showed tumor-targeted accumulation.^227^

### Granulocyte-macrophage colony–stimulating factor (GM–CSF) fusion proteins

GM–CSF, a hemopoietic growth factor, plays an important role in the proliferation and differentiation of hematopoietic cells and is involved in the activation of neutrophils and macrophages as well as the expression of proinflammatory cytokines, adhesion molecules, and costimulatory molecules.^228^ GM–CSF is a monomeric glycoprotein cytokine composed of 127 amino acids forming a four-helix bundle.^229^ GM–CSF has been shown to be a pleiotropic host factor and to be involved in upregulating the expression of antigens,^230,231^ enhancing the production of adhesion molecules on granulocytes^228^ and monocytes and boosting IL-2-induced T cell growth in vitro.^232^ It has been demonstrated that GM–CSF induced tumor necrosis and reduced tumor size in mice vaccinated with irradiated GM–CSF-expressing tumor cells.^233^ In a clinical trial, systemic administration of GM–CSF in melanoma patients enhanced the immune response and greatly contributed to CTL-mediated tumor rejection in vivo.^234^ However, systemic administration of GM–CSF also induced dose-dependent side effects, such as myalgia, fever, fluid retention, and serosal effusion.^235^

GM–CSF was fused to the C-terminus of a scFv targeting the ED-B domain of fibronectin to generate GM–CSF-scFv fusion protein.^236^ Fusion of GM–CSF to the C-terminus of the ch17217 antibody, a rat/mouse chimeric anti-mouse transferrin receptor (TfR) antibody involved in iron uptake, maintained both the function of the antibody and cytokine.^237^ The ch17217-(GM–CSF) fusion protein eliminated hepatic metastases of neuroblastoma NXS2 cells and pulmonary metastases of CT26 colon carcinoma cells in syngeneic A/J and BALB/c mice.^238^ Another fusion protein, ch14.18-(GM–CSF), consisting of human chimeric antiganglioside GD2 antibody 14.18 and GM–CSF, maintained the ability to induce Fc domain-mediated ADCC and CDC activities.^239^ GM–CSF has also been fused to the N-terminus of a scFv fragment of an anti-idiotypic antibody mimicking the TAA CEA.^240^

### IFN-γ fusion proteins

IFN-γ (a type II IFN) is composed of 143 amino acids and contains two N-glycosylation sites. IFN-γ is a head-to-tail dimer, in which one monomer aligns with the N-terminus of another monomer, with a molecular weight of ~100 kDa.^241^ The antitumor activity of IFN-γ depends on its function in the upregulation of class I and class II MHC molecules and the activation of monocytes/macrophages, CD8^+^ T cells, and NK cells. In this regard, IFN-γ is often used in combination with chemotherapy.^242^ In early research, an anti-TAG72 scFv-IFN-γ fusion protein exhibited the antigen-binding specificity of scFv moiety and cytokine activity in vitro.^243^ L19 scFv against the ED-B domain of fibronectin was fused to the N-terminus of a cysteine-free mutant of murine IFN-γ to generate an anti-ED-B-scFv-IFN-γ fusion protein.^242^ The fusion protein targeted blood vessels in solid tumors, and the targeting efficiency was strikingly increased in tumor‐bearing IFN‐γ receptor-knockout mice. scFv(L19)‐IFN‐γ displayed a strong antitumor effect in both subcutaneous and metastatic murine F9 teratocarcinomas. Immunocytokines are usually composed of mAbs against overexpressing antigens (e.g., fibronectin and FAP) in the TME and potent cytokines (e.g., IL-2, IL-12, and GM–CSF). Upon binding to the target antigen, the cytokines are released from immunocytokines directly into the TME, achieving high local concentrations and minimizing systemic side effects.^244^ The protagonists of immunocytokines are cytokines, which share mechanisms similar to that ADC payload release. In recent years, immunocytokines, also known as antibody–cytokine fusion proteins, have attracted increasing attention since the successful application of immune checkpoint antibodies, especially anti-PD1/PD-L1 antibodies. Ideally, in an exemplary immunocytokine context, an immune checkpoint antibody is expected to disable the immune escape mechanism, which is hijacked by tumor cells, and the cytokine payload locally and synergistically regulates the immune response.

At the 2018 American Society of Clinical Oncology conference, Merck KGaA announced an updated clinical study of Bintrafusp alfa (M7824, MSB0011359C), a bifunctional immunocytokine consisting of the anti-PD-L1 antibody Bavencio (avelumab) and the extracellular domain of transforming growth factor-β (TGFβ) type II receptor (TGFR-2).^245^ TGFβ was reported to attenuate the tumor response to PD-L1 blockade by contributing to the exclusion of T cells. Preclinical studies of Bintrafusp alfa revealed that the anti-PD-L1/TGFβ Trap fusion protein, not the anti-PD-L1 antibody, reversed the mesenchymalization of cancer cells, thereby sensitizing tumor cells to chemotherapy (NCT03631706). Furthermore, targeting both PD-L1 and TGF β in the TME is effective for inhibiting immune escape and “warming” cold tumors with limited infiltration of immune cells.^245^ Theoretically, these bifunctional fusion proteins show better clinical benefits than traditional PD1/PD-L1 mAbs. However, a clinical trial (NCT03631706) aimed at evaluating the efficacy of M7824 compared to that of Pabolizumab as a first-class treatment of advanced NSCLC with PD-L1 expression was terminated in January 2021 because it was unlikely to reach a common major endpoint, especially in terms of PFS, according to the collected data (Table 5). To date, antibodies have been fused not only with cytokines but also with other immune checkpoint targets; for example, Cinrebafusp alfa is the fusion of an anti-HER2 antibody with 4–1BB, an immune checkpoint protein, to generate the FAP-targeted 4–1BB agonist (FAP-4–1BBL). In China, I-Mab Biopharma developed the fusion protein efineptakin (also called TJ107), which is composed of an anti-PD-1 antibody and IL-7, and this protein was entered into a 2019 phase Ib clinical trial.

**Table 5:** Clinical trials of Bintrafusp alfa (M7824)

ClinicalTrials.gov identifier	Description	Indication(s)	Status
NCT03840915	In combination with chemotherapy	Stage IV non-small cell lung cancer	Phase Ib/II
NCT03840902	M7824 vs. durvalumab	Unresectable stage III non-small cell lung cancer	Phase II
NCT03833661	M7824 monotherapy	Locally advanced or metastatic second line (2L) biliary tract cancer (cholangiocarcinoma and gallbladder cancer)	Phase II
NCT03631706	M7824 vs. Pembrolizumab	A first-line (1L) treatment in participants with programmed death-ligand 1 (PD-L1) expressing advanced non-small cell lung cancer (NSCLC)	Phase II
NCT02517398	M7824	Metastatic or locally advanced solid tumors	Phase I
NCT02699515	M7824	Metastatic or locally advanced solid tumors	Phase I

Cytokines can activate the immune system to achieve antitumor effects. Some cytokines, such as IL-2, have been authorized for the treatment of certain types of malignancies.^246^ However, dose-dependent side effects and systemic toxicity have limited the clinical applications of cytokines. Immunocytokines link cytokines to antibodies specific to TAAs, similar to BsAbs. Antibodies targeting TAAs or tumor-specific antigens (TSAs) can deliver cytokines to tumor sites where they can locally exert their antitumor effects.

## Antibody fragments and non-Ig scaffold proteins

### Fab

**Fig. 6:**
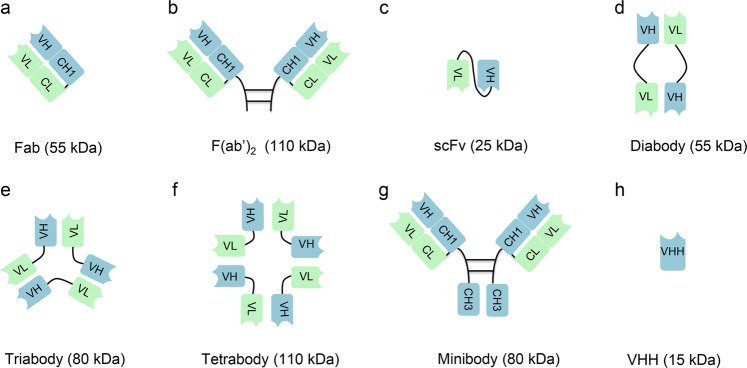
Different formats of antibody fragments and their derivatives

A Fab is a monovalent fragment consisting of one light chain and one heavy chain linked by a disulfide bond (Figs. 1b and 6a). Many in vivo experiments have shown that diffusion plays an important role in transporting molecules into tumor tissues.^247^ With a molecular weight of ~55 kDa, a Fab is much smaller than full-size IgG and thus shows greater penetration in solid tumors.^248^ For example, Lucentis, a VEGF-A-specific Fab fragment was more effective than the corresponding full-length mAb Avastin, in treating patients with the wet form of macular degeneration.^24^ Lacking the Fc region, a Fab can be produced in an economic expression system (e.g., yeast) and prevent Fc-related adverse effects in vivo. Predictably, a Fab has a shorter half-life due to the lack of FcRn-mediated recycling, similar to a scFv.^249^ The Fab half-life can be easily extended by conjugation to PEG or fusion with an albumin-binding protein.^250^ The FDA has approved certolizumab pegol (Cimzia®), a PEGylated and humanized anti-TNFα Fab, for the treatment of Crohn’s disease and rheumatoid arthritis.

Fab was considered the first therapeutic fragment antibody format, and eight Fab fragment antibodies were entered into clinical trials in the mid-1990s.^251^ To date, three Fabs have been approved by the FDA: abciximab (Reopro®), idarucizumab (Praxbind®), and ranibizumab (Lucentis®).^250^ No Fab has been approved for cancer treatment. Notably, naptumomab estafenatox (5T4FabV18-SEA/E-120 or ABR-217620), a fusion protein consisting of an anti-5T4 Fab and superantigen-staphylococcal enterotoxin A (SEA), was reported to successfully improve the OS of renal cell carcinoma patients in phase II/III trial.^252,253^

### F(ab’)_2_

F(ab’)_2_, with a molecular weight of 110 kDa, is a bivalent fragment composed of two Fab segments linked together by a hinge region (Figs. 1b and 6b). They show better penetration than mAbs due to their smaller size.

The China FDA approved Metuximab-I131, a radioactive iodine [^131^I]-labeled anti-CD147 F(ab’)_2_ mAb, for the treatment of liver cancer. No F(ab’)_2_ has been approved by the FDA for cancer therapy.

### Single-chain variable fragment (scFv)

The scFv was first described by Bird et al. in 1988.^254^ The scFv antibody consists of variable regions of heavy (VH) and light (VL) chains, which are joined together by a flexible peptide linker (e.g., GGGGS), and the biologically active form of scFv can be easily expressed in *Escherichia coli*, dramatically reducing production costs (Figs. 1b and 6c).^255^ The molecular weight of an scFv is ~25 kDa, which is much smaller than that of a full-length mAb, thus showing penetration in tumors. The use of an scFv can prevent Fc-related side effects resulting from ADCC or CDC.

However, the lack of the Fc region causes faster renal clearance (half-life ~5 h) and poor stability of the scFv, which tends to aggregate and show high immunogenicity.^24,249^ The multimerization of scFvs is a viable strategy to improve the pharmacokinetic properties and affinity of scFvs, such as diabodies (bivalent dimers, 55 kDa),^256^ triabodies (trivalent dimers, 80 kDa),^257^ tetrabodies (tetravalent dimers, 110 kDa),^258^ and minibodies (80 kDa).

Clinical trials of scFv antibodies have been conducted since the first scFv-focused clinical study performed in 1995.^259^ For example, several monovalent scFv fusion proteins are under development in clinical trials for cancer therapy, including L19-IL-2, rM28, D2C7-IT (in phase I), and Vicinium (in phase III). Multivalent or multispecific scFvs, such as BiTE, are discussed in the section on multispecific antibodies.

The leading scFv drug Vicinium (Sesen Biotech) is a humanized single-chain antibody fragment specific to the EpCAM antigen fused with Pseudomonas exotoxin A (ETA) (252–608 aa) toxin and was designed for treating bladder cancer. Upon binding with the EpCAM antigen on the surface of cancer cells, Vicinium can be internalized through the endocytic pathway and release the toxin ETA (252–608), thereby inducing cell apoptosis by irreversibly blocking protein synthesis.

### VHH domain

In 1993, Hamers–Casterman discovered a special antibody in camelid that lacks a light chain and heavy chain CH1 domain, so it was called a heavy chain antibody (HCAb) (Figs. 1b and 6h).^260^ Later, HCAbs were found in Camelidae (bactrian and dromedary camels, alpacas, and llamas), as well as cartilaginous fish (e.g., sharks, rays, and skates).^261^ Interestingly, the variable domain of the heavy chain in a HCAb (also known as the VHH domain, a nanobody or single-domain antibody), despite a molecular weight (~15 kDa) one-tenth that of IgG (~150 kDa), retains high antigen-binding affinity, and is therefore the smallest naturally derived antigen-binding fragment.^261^ A crystal structure assay of the VHH domain revealed that its dimension is 4 nm × 2.5 nm × 3 nm.^262^

The homology discovered between the VHH and V_H_ domains in the VH III human Ig family was greater than 80%.^263^ This means that the VHH sequence may induce relatively low immunogenicity when applied to cancer immunotherapy.^264^

VHH domains were demonstrated to be highly soluble and more stable than conventional antibodies and are capable of being stored for more than months at 4 or −20 °C without losing notable antigen-binding capacity.^265^ VHH domains can survive under very harsh conditions, for example, a wide pH range (range 3.0–9.0) and chemical (e.g., 6–8 M urea treatment) and thermal denaturing conditions (e.g., a VHH domain can maintain antigen-binding activity after prolonged incubation at 90 °C),^265^ making various routes, intravenous, oral, intraperitoneal or intratumor injection, feasible.^266^ Importantly, a VHH domains have a completely hydrophilic surface, which accounts for their superior stability and solubility compared to that of IgG VH domains, and VHHs show significantly less aggregation during production or multimerization (e.g., tandem VHH-based multispecific antibodies).^265^

In addition, the CDR3 loop in camelid VHH domains is usually longer (3–28 amino acids) than conventional VH domains in human IgG (8–15 amino acids).^267^ The long CDR3 of a VHH, which determines its recognition specificity, expands the potential interaction surface with a target antigen in the absence of a VL domain.^265,268^ Interestingly, the longer CDR3 in VHH domains can form a finger-like appendage that fits into a protein cleft, enabling the recognition of epitopes that are not accessible to the larger mAbs.^267^

The small size of the VHH domain results in fast renal clearance (half-life ~2 h),^269^ which is a major disadvantage for the application of VHH domains in cancer treatment but an advantage for obtaining an optimal signal-to-noise ratio during its application as an in vivo imaging agent.^270^ VHH domains have recently emerged as promising targeted imaging probes in combination with traditional imaging techniques, such as positron emission tomography (PET)^271^ and single-photon emission computed tomography (SPECT).^272^ The most noteworthy case of a VHH domain-based probe used for in vivo tumor imaging is the 68Ga-NOTA anti-HER2 VHH, which is a radiolabeled VHH domain currently in phase II clinical trial being evaluated for its ability to detect brain metastasis in breast cancer patients via PET/CT (NCT03331601).

Recently, the FDA and EMA approved the first VHH drug, caplacizumab (Ablynx Inc), a humanized anti-VWF VHH domain used for treating adults with acquired thrombotic thrombocytopenic purpura. A variety of VHH domains are being studied in the clinic to assess their effects for different purposes, including cancer treatment; for example, anti-EGFR VHH,^36,273^ anti-HER2 VHH,^274^ anti-VEGFR2 VHH,^275,276^ anti-c-Met VHH,^6,277^ and anti-chemokine receptor type 7 (CXCR7) VHH^278^ are currently being evaluated. Two VHH domains are in phase I clinical trials for cancer therapy; these VHH-based drugs are a biparatopic anti-CXCR4 VHH ALX-0651 (Ablynx, Inc.) for use in stem cell mobilization and novel agonistic tetravalent anti-DR5 VHH TAS266 (Novartis) used against advanced solid tumors. In its phase I clinical trial, TAS266 was found to show unexpected and significant but reversible hepatotoxicity, which can probably be attributed to enhanced DR5 clustering and activation of hepatocyte apoptosis.^279^ Tables 6 and 7 summarize the antibody fragments that have been approved for use by the FDA and those that have been entered into a clinical trial. The formats of presentative antibody fragments and their derivatives are summarized in Fig. 6.

**Table 6:** The FDA-approved antibody fragments

Generic (brand) name	Format	Target	Indication(s)	Sponsor	Year of FDA approval
Ranibizumab/Lucentis	Humanized Fab	VEGF	Neovascular (wet) age-related macular degeneration	Gentech	2006
Abciximab/ReoPro	Chimeric Fab	GPIIb/IIIa	Cardiovascular	Centocor	1994
Certolizumab pegol/Cimzia	PEGylated humanized Fab’	TNF-α	Crohn’s disease, rheumatoid arthritis	UCB	2008
Idarucizumab/Praxbind	Humanized Fab	Dabigatran	Anticoagulation	Boehringer-Ingelheim	2015
Digibind	Ovine Fab	Digoxin	Digoxin overdose	GlaxoSmithKline	1986
DigiFab	Ovine Fab	Digoxin	Digoxin overdose	Protherics	2001
CroFab	Ovine Fab	Crotalidae venom	Pit viper envenomation	Protherics	2000
Anavip	Equine F(ab’)_2_	Crotalidae venom	Pit viper envenomation	Rare Disease Therapeutics	2015
Anascorp	Equine F(ab’)_2_	Centruroides venom	Arizona bark scorpion envenomation	Rare Disease Therapeutics	2011
Brolucizumab/Beovu	Humanized scFv	VEGF	Neovascular (wet) age-related macular degeneration (AMD)	Novartis	2019
Caplacizumab/Cablivi	Humanized VHH	vWF	Acquired thrombotic thrombocytopenic purpura (aTTP)	Ablynx	2019

**Table 7:** The clinical-stage antibody fragments and their derivatives for cancer treatment

Name	Format	Target	Indication(s)	Status	ClinicalTrials.gov identifier
Copper Cu 64-DOTA-B-Fab	Fab	CA6	Ovarian carcinoma Breast carcinoma	Phase I	NCT02708511
CSR02-Fab-TF	Fab	PLVAP	Hepatocellular carcinoma (HCC)	Early Phase I	NCT04601428
Ranibizumab	Fab	VEGF	Uveal melanoma	Phase IV	NCT00540930
Naptumomab estafenatox	Fab and SEA fusion protein	5T4	Renal cell carcinoma	Phase II Phase III	NCT00420888
IMCgp100	Monoclonal TCR anti-CD3 scFv fusion protein	CD3	Advanced metastatic melanoma	Early Phase I	NCT01209676
L19-IL2	Recombinant scFv	EDB	Solid tumor	Phase I	NCT02086721
rM28	Bispecific scFv	CD28/HMV-MAA	Malignant melanoma	Phase I	NCT00204594
D2C7-IT	scFv-based immunotoxin	EGFRwt and EGFRvIII	Recurrent malignant glioma	Phase I	NCT02303678
NM21–1480	Trispecific scFv fusion protein	PD-L1/4–1BB/HSA	Advanced solid tumor	Phase I Phase II	NCT04442126
Vicinium	scFv-based immunotoxin	EpCAM	Bladder cancer	Phase III	NCT02449239
[124 I] PSCA-Minibody	Minibody	PSCA	Prostate cancer Pancreatic cancer Bladder cancer	Phase I	NCT02092948
6B11-OCIK	Minibody	6B11	Recurrent platinum-resistant ovarian cancer	Phase I	NCT03542669
T84.66	Iodine I 123 anti-CEA recombinant diabody	CEA	Colorectal cancer	Phase I	NCT00647153
BCMA VHH CAR-T Cell	VHH	BCMA	Relapsed/refractory myeloma	Phase I	NCT03664661
CD19/20 bispecific VHH-derived CAR-T Cells	VHH	CD19/CD20	Refractory/relapsed B cell lymphoma	Phase I	NCT03881761
ALX-0651	VHH	CXCR4	Healthy volunteers	Phase I	NCT01374503
αPD1-MSLN-CAR T cells	Secreting PD-1 VHHs	MSLN	Non-small cell lung cancer Mesothelioma	Early Phase I	NCT04489862
			Colorectal cancer Ovarian cancer		NCT04503980
[131I]-SGMIB anti-HER2 VHH1	VHH	HER2	Healthy volunteers Breast cancer	Phase I	NCT02683083
68-Ga NOTA-anti-MMR-VHH2	VHH	MMR	Malignant solid tumor Breast cancer Head and neck cancer Melanoma (skin)	Phase I/IIa	NCT04168528
68-GaNOTA-anti-HER2 VHH1	VHH	HER2	Metastatic breast carcinoma Locally advanced breast cancer	Phase II	NCT03924466
			Breast neoplasm Breast carcinoma Receptor, ErbB-2	Phase II	NCT03331601
99mTc-MIRC208	VHH-based radiotracer	HER2	HER2-positive cancer	Preclinical	NCT04591652
TAS226	Tetravalent VHH	DR5	Advanced solid tumors	Phase I	NCT01529307

### Affibody

One key priority in current studies is the generation of homogeneous BsAbs to overcome hybrid pairing of heavy and light chains derived from two antibodies expressed in the same cell.^280^ Utilizing non-Ig scaffolds as building blocks is a viable approach to circumventing this problem.^280^ Non-IgG proteins include affibodies,^281^ anticalins,^282^ DARPins,^283^ monobodies,^284^ and so on.

Non-IgG protein scaffolds are typically composed of a single polypeptide chain folded into a structured core,^280^ which is much smaller than an antibody. The large size of an IgG antibody limits its penetration and infiltration in tumor tissues. The attractive characteristics of non-IgG proteins include but are not limited to their small size, high thermal stability, high solubility, the inclusion of a cysteine-free sequence and storage stability.^285^

Affibodies constitute a class of binding proteins based on the scaffold Z-domain, which is derived from domain B of staphylococcal protein A (*SPA*).^281^ An affibody is composed of a single polypeptide containing 58 amino acid residues with a molecular weight of approximately 6.5 kDa. The substitution of a glycine residue^29^ with an alanine residue in the B domain confers high stability and rapid folding to the Z-domain through the stabilization of helix 2.^286^ Therefore, affibodies are characterized by small size, high stability, and rapid and independent folding of the Z-domain structure.^287^ The combinatorial randomization of 13 amino acid residues in the Z-domain surface (helices 1 and 2), determined on the basis of the X-ray crystallography complex structure obtained for the homologous B-domain of *SPA* and human IgG, has led to the generation of affibody molecule libraries for antigen-binding screening.^288^ Affibodies have advantageous features for therapeutic applications, such as (i) easy production in *E. coli* or through solid-phase peptide synthesis due to their robust physical properties, including stability, fast folding, and ability to withstand a wide range of pH values and temperatures; (ii) small size resulting in rapid tissue penetration and efficient delivery of high molar doses compared to larger proteins; (iii) high affinity (with a *K*_D_ of ~pM) for the target protein with nonspecific binding^289^; and (iv) site-specific conjugation facilitated by a unique C-terminal cysteine residue can be performed. Affibodies show the most promise as imaging probes due to their high tumor retention and tissue penetration, rapid blood clearance kinetics, low uptake by nontumorous organs, and the ability to undergo rapid, site-specific labeling with different radionuclides depending on the preferred modality.^290^ In vivo imaging studies using affibody proteins mainly focus on targeting HER2^291^, which is overexpressed in breast carcinomas and a validated target for antibody-based immunotherapy.

Affibody molecules, which is a rapidly growing class of non-IgG affinity ligands, show some advantages over antibodies. In addition to their potential in vivo imaging, affibodies can be applied in therapeutics. In 2020, a bispecific affibody (ABY-035) targeting subunit IL17A and human albumin for the extension of serum half-life was developed to treat patients with psoriatic arthritis (PsA), and the results from a phase I trial demonstrated that ABY-035 was safe and well tolerated (NCT03591887).

Another group of non-IgG scaffold proteins consists of anticalins, which are composed of a rigid β-barrel with four exposed loops that are engineered binding proteins based on the natural lipocalin fold.^282^ The central β-barrel supports four structurally variable loops that form a binding site.^292^ According to X-ray structural analysis, reshaping these loops can lead to the anticalin ability to recognize and tightly bind a wide range of molecules, from small molecules to peptides and proteins.^292^ The rigid and small structure of the non-IgG scaffold proteins enables flexible protein fusion and chemical conjugation with or incorporation into multifunctional molecules, such as multispecific antibodies and ADCs.^280^

A modified anticalin specifically binds to fibronectin ED-B,^293^ which can be used for the diagnosis of glioblastomas.^294^ ED-B-specific anticalins are located in the blood vessels of a glioblastoma, especially in the endothelial cells of glioblastoma origin.^294^ On the other hand, anticalins can be used to construct BsAbs for binding targets. For example, bispecific duocalin generated from two anticalins recognizing fluorescein and digoxigenin, respectively, has been successfully applied to fluorescence titration experiments.^295^

DARPins are scaffold proteins engineered on the basis of human ankyrin repeat proteins.^283^ DARPins are composed of 33 amino acid residues that form tightly packed repeats.^296^ Two antiparallel α-helices following a β-turn in each repeat form a structural unit.^297^ A right-handed solenoid structure with a continuous hydrophobic core constitutes four to six repeats in each ankyrin repeat domain.^298^ Currently, DARPins are applied in many fields, for example, as selective inhibitors of c-Jun N-terminal kinase 1 (JNK1) or JNK2.^299^ An EpCAM-specific soluble DARPin was produced as a fusion protein with *Pseudomonas aeruginosa* exotoxin A (ETA) in *E. coli.*^300^ This DARPin-ETA fusion protein was found to be highly toxic to EpCAM-positive tumor cells and exhibited strong antitumor efficacy in a mouse xenograft model.^300^

Monobodies are synthetic scaffold proteins based on the fibronectin type III (FN3) domain in human fibronectin.^284^ Monobodies can strongly bind to an epitope on the desired target^301^ to disrupt the biological function of the target molecule or protein.^284^ Monobodies have no cysteine residues with which to form intrachain disulfide bonds, and therefore, their functions are not influenced by the reducing environments of intracellular compartments.^284^ The SH2-kinase interaction is necessary for leukemogenesis, and intracellularly expressed monobodies targeting SH2-kinase can inhibit leukemia cell survival and oncogenic transformation.^302,303^ RAS and its mutants remain the most challenging drug targets.^304^ NS1, a monobody identified by the unbiased selection, binds to KRAS and HRAS by recognizing the uncharacterized α4/α5 region located on the opposite side of RAS.^305^ However, NS1 did not inhibit the GAP, GEF, or other downstream effectors of RAS.^306^ This evidence indicated that NS1 disrupted the formation of a signaling complex containing two RAS molecules and two RAF kinase molecules by inhibiting RAS self-association.^284^

The discovery of agents capable of disrupting protein–protein interactions is one of the major goals of the biopharmaceutical industry aimed at therapeutics development.^307^ These non-IgG scaffold proteins have been advanced in mechanistic studies and used to identify prospective therapeutic target drug-binding sites.

### TCRm antibody

Most cancer-specific targets are intracellular proteins that are inaccessible to traditional mAbs.^308^ Intracellular proteins can be degraded and processed into peptides by the proteasome and ultimately presented on the cell surface in the context of MHC class I molecules for recognition by TCRs on CD8^+^ T cells.^309^ Although TCRs can bind target peptide-loaded MHC molecules specifically, their application as therapeutic agents has been limited by a low binding affinity for pMHC (1–100 µM)^310^ and poor yield during production in expression systems.^311^ It is feasible to stabilize TCRs computationally via rationally designed mutagenesis,^312^ but TCR affinity maturation and TCR expression remain challenging. An alternative strategy for targeting pMHC is the development of TCRm mAbs (Fig. 1a). Compared with TCR antibodies, TCRm antibodies show greater affinity (100–1000×) for pMHC and maintain all the advantages of IgG antibodies, such as stability, high yield, and a well-established production system.^30^ In addition, anti-TCRm mAbs can be generated efficiently via animal immunization and hybridoma technology or through in vitro display technologies, e.g., phage and yeast displays.^313^

In contrast to traditional mAbs, whose targets are mainly cell surface antigens (~10% of their total targets), TCRm mAbs provide a viable strategy to target the ~90% remaining intracellular antigens through pMHC.^314^ The targets of TCRm mAbs are classified into two types: TAAs and neoantigens, which are also known as tumor-specific antigens. Based on the origin of intracellular proteins, TAAs are classified into tumor-associated viral antigens and tumor-associated self-antigens. In some virus-induced cancers, viral proteins (e.g., CMV proteins^315^ and EBV proteins^316^) can be degraded and presented on the cell surface. They are referred to as tumor-associated viral antigens. Tumor-associated self-antigens are normal proteins that are abnormally expressed in tumor cells, including cancer-testis antigens, oncofetal antigens, differentiation antigens, and overexpressed antigens, e.g., Wilms tumor protein (WT1),^317^ glycoprotein (gp100), melanoma antigen (MAGE),^318^ melanoma-associated antigen recognized by T cells-1 (MART-1),^319^ and NY-ESO-1.^320^ On the other hand, neoantigens are results of tumor-specific somatic missense mutations, such as Kras G12V/D^321^ and p53.^322^ With respect to the high level of tumor specificity, the aforementioned pMHC antigens are emerging as conceptually ideal targets for targeted therapies prevent on-target off-tumor toxicity.^323^

The average low density of pMHC antigens appears to be the major hurdle for using TCRm mAbs in clinical application. Various strategies have been applied to enhance the potency of TCRm mAbs whose efficacy and affinity can be significantly affected by the low expression level of pMHC. One strategy is to enhance the Fc region functionalities, such as those that induce ADCC, CDC, and ADCP. ESK1 is a TCRm mAb that specifically recognizes the WT1 RMF/HLA-A*02:01 complex.^324^ To enhance the efficacy of ESK1, Nicholas Veomett et al. altered its Fc glycosylation to obtain a glycoengineered TCRm mAb, “ESKM.” As a result, ESKM exhibited potent ADCC activity at lower doses and superior in vivo efficacy than the parental antibody ESK1.^325^

The potency of TCRm mAbs can be enhanced by transforming TCRm mAbs into ADCs, BsAbs, or CARs. After equipping them with highly cytotoxic payloads, TCRm mAbs confer additional cytotoxicity, and thus, they can specifically deliver cytotoxic payloads into target tumor cells. In 2008, Klechevsky, E. et al. first fused TCRm antibodies targeting MART-1_26–35_/HLA-A*02:01 and gp100_280–288_/HLA-A*02:01 with a truncated form of Pseudomonas exotoxin to generate a TCRm immunotoxin with antitumor activity.^326^ Our group developed EA1 HL-vcMMAE, a TCRm-ADC-targeting MART-1_26–35_/HLA-A*02:01, for the treatment of metastatic melanomas, and we found that it showed potent in vivo efficacy in a mouse xenograft model.^327^ Recently, we generated a novel TCRm ADC 2A5-MMAE against the neoantigen Kras G12V/HLA-A*0201, and it showed specific antitumor activity both in vitro and in vivo.^328^ However, the potency of TCRm ADCs has been limited by extremely low levels of cell surface pMHC density compared with ADCs against normal targets. In another validated strategy, TCRm mAbs are applied as tumor-binding modules in the context of BsAbs to redirect and mediate T cells to specifically kill tumor cells. For example, the same group that discovered ESK1 engineered ESK1 into a BiTE.^329^ Despite the extremely low density of WT1/HLA-A*02:01 on the cell surface, ESK1-BiTE showed potent efficacy against multiple leukemias and solid tumors in vitro and in vivo. Interestingly, ESK1-BiTE also induced a robust secondary T cell response on the basis of its high specificity for HER2/Neu epitope 369 in an autologous in vitro setting. Thus, an epitope-spreading effect can potentially be another ESK1-BiTE mechanism of action. A TCRm can also be engineered into a TCRm CAR to redirect T cells.^330^ An ongoing early phase I clinical trial is aimed at evaluating GPA-TriMAR-T cells, which are TCRm-CAR-T cells targeting gp100_209–217_/HLA-A*0201, in patients with malignant melanoma (NCT03649529).

Drug combinations can improve therapeutic efficacy. FDA-approved TKIs are not effective in patients with Ph^+^ ALL. Since high expression levels of WT1 RMF/HLA-A*02:01 are found in Ph^+^ ALL, a TCRm mAb ESKM alone or in combination with TKIs was used in patients with Ph+ ALL. The results showed that ESKM alone is more effective than TKIs. Furthermore, the combination therapy with ESKM and TKIs showed superior efficacy than monotherapy with only TCRm ESKM or TKIs.^331^ Many small-molecule agents, such as proteasome inhibitors, histone deacetylase inhibitors, and MEK inhibitors, are capable of upregulating MHC expression and presentation, which facilitate pMHC-targeted tumor therapy.^332^ For example, our previous study showed that the MEK inhibitor trametinib augmented the antitumor efficacy of EA1 HL-vcMMAE both in vitro and in vivo by increasing MART-1_26–35_ peptide presentation.^328^

In conclusion, TCRm mAbs, which combine the specificity of TCR recognition with the favorable properties of antibodies, have emerged as promising therapeutics, particularly in cancer treatment. Despite their potential for killing tumor cells specifically, clinical trials to evaluate TCRm mAbs are still pending. Future development of TCRm mAbs will focus on identifying druggable neoantigens and addressing challenges of TCRm mAbs application, including inadequate antigen presentation, lack of MHC internalization, and cross-reactivity with other epitopes.^332^

## Conclusions and future perspectives

Recently, new formats of therapeutic antibodies have been described, and studies on traditional non-IgG immunoglobins, such as IgA and IgM, are undergoing a renaissance.^333,334^ Here we summarize the advances of new-format therapeutic antibodies in cancer therapy, including antibody conjugates (e.g., ADCs, AOCs, and radiolabeled antibodies), bispecific/multispecific antibodies, immunocytokines, antibody fragments (e.g., Fabs, scFvs, and VHH domains), and scaffold proteins. Miniaturization and multifunctionalization represent two major directions in antibody development. As described in this review, full-length antibodies have been transformed into fragments, e.g., Fabs, scFvs, and VHH domains, and small scaffold proteins (e.g., affibodies and DARPins) have been rationally designed to enhance tumor penetration and facilitate fast serum clearance, which are advantages for their applications in tumor diagnostic imaging. Radiolabeled VHH domains are promising in vivo imaging probes when combined with traditional imaging techniques (PEG/SPECT).^335^ Moreover, the small size and robustness of antibody fragments show superior local performance, for example, eye injection of ranibizumab (Lucentis, an anti-VEGF Fab) and the subcutaneous injection of sonelokinab (an anti-IL-17 A/F VHH).^336,337^

The multifunctionalization of therapeutic antibodies includes antibody derivatives (e.g., ADCs and AOCs) and antibody fusion proteins (e.g., multispecific antibodies and immunocytokines). ADCs and AOCs utilize payload-induced cytotoxicity and oligonucleotide functionality, respectively, in combination with the exquisite targeting ability of the antibody to show improved biodistribution profiles, attacking tumor cells via multiple mechanisms of action. An Fc-enhancing technique, such as P329G LALA mutation for ADCC enhancement and E430G for CDC enhancement, can further increase the potency of multifunctionalized antibodies in the treatment of solid tumors.^338,339^ Multispecific antibody-based strategies have been extensively explored to leverage the local TME in an antigen-dependent manner, such as T cell costimulation, engagement of innate and adaptive immune cells, simultaneous blockade of two immune checkpoints, and targeting multiple antigens to increase tumor selectivity. The poor infiltration of immune effector cells and complex immunosuppressive TME in solid tumors requires combination therapies consisting of multispecific antibodies with other immunomodulatory agents, for example, immune checkpoint blockers, personalized neoantigen vaccines, and oncolytic virus.^161^

Soluble TCRs, TCRm antibodies, and their derivatives (e.g., ImmTAC molecules) can recognize highly tumor-specific HLA-restricted peptides (e.g., p53 and KRAS), which are considered undruggable targets of therapeutic antibodies.^340,341^ The rapid development of second-generation sequencing, single-cell RNA sequencing, spatial omics, and integrated bioinformatics analysis enables an in-depth and comprehensive understanding of malignant tumor tissues and will lead to an era of antibody-based intracellular antigen targeting and precise tumor therapy.
